# Nucleotide composition shapes gene expression in *Wolbachia pipientis*: a role for MidA methyltransferase?

**DOI:** 10.1128/msystems.00779-25

**Published:** 2025-08-15

**Authors:** Stella Papaleo, Simona Panelli, Ibrahim Bitar, Lodovico Sterzi, Riccardo Nodari, Francesco Comandatore

**Affiliations:** 1 Department of Biomedical and Clinical Sciences, Pediatric Clinical Research Center "Romeo and Enrica Invernizzi," University of Milan 9304, Milan, Italy; 2 Biomedical Center, Faculty of Medicine, Charles University 60569 , Pilsen, Czechia; 3 Istituto Nazionale di Genetica Molecolare (INGM) "Romeo and Enrica Invernizzi," University of Milan 9304, Milan, Italy; The Pennsylvania State University, State College, Pennsylvania, USA

**Keywords:** *Wolbachia pipientis*, MidA, gene expression, regulation of gene expression, endosymbionts

## Abstract

*Wolbachia pipientis* is an obligate intracellular bacterium, associated with several arthropods and filarial nematodes. *Wolbachia* establishes a variety of symbiotic relationships with its hosts, with consequent genomic rearrangements, variation in gene content, and loss of regulatory regions. Despite this, experimental studies show that *Wolbachia* gene expression is coordinated with host developmental stages, but the mechanism is still unknown. In this work, we analyzed published RNA-seq data of four *Wolbachia* strains, finding a correlation between gene nucleotide composition and gene expression. The strength and direction of this phenomenon changed with the expression of the S-adenosyl-methionine-dependent methyltransferase *mid*A. Specifically, when *mid*A is overexpressed, there is a negative relationship between gene adenine content and gene expression, while downregulation of *mid*A reverses this trend. MidA is known to methylate protein arginine, with potential effect on protein affinity for substrates, including nucleic acids. To expand our understanding of this poorly characterized enzyme, we investigated its ability to methylate DNA expressing it in *Escherichia coli*. The experiment revealed that the *Wolbachia* MidA can methylate both adenine and cytosine. Lastly, we found upstream the *mid*A gene, a conserved binding site for the Ccka/CtrA signaling transduction system, and we hypothesize that this mechanism could be involved in the communication between the host and the bacterium. Overall, these findings suggest a cascade mechanism in which the host activates the bacterium Ccka/CtrA signaling system, thus inducing the expression of the midA gene, with subsequent effect on the expression of several *Wolbachia* genes on the basis of their nucleotide composition.

**IMPORTANCE:**

*Wolbachia pipientis* is one of the most common intracellular bacteria in insects, and it is currently utilized as a tool for the control of vector-borne diseases. As for many other endosymbiont bacteria, *Wolbachia* experienced important genome rearrangements, gene content changes, and the loss of several regulatory sequences, affecting the integrity of operons and promoters. Nevertheless, experimental studies have shown that *Wolbachia* gene expression is coordinated with the host physiology (e.g., developmental stages), although the underlying mechanism remains unclear. In this work, based on *in silico* analyses and an experimental study on wOo methyltransferase, we propose that bacterial DNA methylation could be a key mechanism regulating *Wolbachia* gene expression. Additionally, we found evidence suggesting that the DNA methylation process in *Wolbachia* can be activated by the host.

## INTRODUCTION

*Wolbachia pipientis* (from here *Wolbachia*) is an intracellular bacterium belonging to the Alphaproteobacteria class. It is the most prevalent endosymbiotic microbe in the animal world, colonizing many arthropod species and filarial nematodes (1–4). In arthropods, the bacterium can manipulate the reproduction of the host (5, 6), protect it from viruses (7, 8), and provide nutrients (9). In filarial nematodes, genomic analyses and experimental evidence revealed that *Wolbachia* is essential for the host survival and development (10). It is mainly mother-to-offspring transmitted, in some cases being actively transported by specialized cells of the host from the soma to germ cells (2). Nevertheless, horizontal transfer among host individuals can occur (11, 12), enhancing the bacterium spreading (12).

*Wolbachia* genus comprises several lineages with ecological and physiological specificities. As for many other strictly host-associated bacteria, the genome of *Wolbachia* strains shows signs of important reduction during their evolution, reaching a size that ranges from ~0.8 to ~1.8 Mb (data from the database Bacterial and Viral Bioinformatics Resource Center - BV-BRC) (13). As observed for several endosymbiont bacterial species, genome reduction passes through the increasing of the number of mobile elements (e.g., insertion sequences) and the change in gene content (14–16). During this process, the structure of the genome and the order of genes can change dramatically (15), leading to the disruption of several operons and loss of most gene promoters (17). Interestingly, RNA-seq studies have shown that *Wolbachia* gene expression is coordinated with the physiology of the host (18–24), a phenomenon similar to what is observed in another endosymbiont bacteria, *Buchnera aphidicola* (25). This implies that *Wolbachia* may possess mechanisms for regulating gene expression, even though, as in other endosymbionts, the number of promoters is expected to be low (17). These findings suggest that regulation may occur through mechanisms beyond traditional transcription factors.

Two mechanisms expressed by *Wolbachia* and possibly involved in its gene regulation are the PleC/PleD and CckA/CtrA systems (26). The PleC/PleD system consists of the transmembrane histidine kinase PleC and the response regulator PleD. When PleC is activated, it phosphorylates PleD, which in turn catalyzes the synthesis of c-di-GMP from GTP. The CckA/CtrA system consists of a membrane-bound receptor (CckA) and a cytoplasmic response regulator (CtrA). Upon activation by an external signal, CckA phosphorylates CtrA, which then binds to the specific DNA sequence TTAA-N7-TTAA upstream of target genes to induce their expression. When triggered by an external stimulus, the Ccka protein activates CtrA, which in turn binds to the CtrA-binding site initiating the expression of the downstream genes. While the signaling mechanisms behind the activation of Ccka are not fully understood, the downstream response regulator, CtrA, is a well-described master regulator with transcription factor activity, conserved in Alphaproteobacteria (27). In various bacteria, this system regulates processes, such as cell division, DNA replication, and differentiation. In *Wolbachia*, it is hypothesized that this mechanism also plays a role in regulating gene expression, particularly during chromosome replication (28).

Christensen and Serbus (26) analyzed seven complete *Wolbachia* genomes, discovering only 34–55 open reading frames (ORFs) having a CtrA-binding site in the 450 bases upstream of the start of translation. This result suggests that, despite CtrA being a pivotal actor of gene expression regulation in *Wolbachia*, other mechanisms can be involved.

The first published *Wolbachia* RNA-seq study (18) compared gene expression of *Wolbachia* colonizing somatic tissues and gonads in females and males of the filarial nematode *Onchocerca ochengi*. One of the strongest differentially expressed genes was an S-adenosyl-methionine-dependent (SAM)-dependent arginine methyltransferase, called mitochondrial dysfunction A or *mid*A. Arginine methyltransferases catalyze the methylation of the nitrogens in arginine residues—a key post-translational modification that is widespread across organisms. Indeed, *mid*A ortholog genes are found in both eukaryotes and prokaryotes (29), with the human ortholog known as NADH ubiquinone oxidoreductase complex assembly factor 7. The function of this methyltransferase has been experimentally studied in humans and in the amoeba *Dictyostelium discoideum* (30, 31), while its function in prokaryotes has been only inferred *in silico* (29, 32). In humans and *D. discoideum*, MidA enzyme participates in the assembly of mitochondrial complex I. In particular, MidA methylates the NADH:ubiquinone oxidoreductase core subunit S2 (NDUFS2) substrate, enabling in turn its binding with NADH:ubiquinone oxidoreductase core subunit S7 (NDUFS7). Indeed, arginine methylation can significantly alter a protein affinity for substrates, including other proteins (e.g., NDUFS2 and NDUFS7) or nucleic acids (32).

In this study, we explore a possible mechanism for gene expression regulation in *Wolbachia*. Initially, we re-analyzed the RNA-seq data from published studies to examine whether the frequency of nucleotides and other composition parameters in genes correlates with their expression. We also found an association between the expression of the *midA* gene and the strength of the correlation between nucleotide composition parameters and gene expression. Lastly, we found a CtrA-binding site conserved among *Wolbachia* strains upstream of the *mid*A gene.

Our results suggest that the host may specifically induce the expression of the *Wolbachia mid*A gene through the Ccka/CtrA signaling transduction system. Subsequently, the MidA methyltransferase may alter gene expression on the basis of their nucleotide composition, possibly acting through an epigenetic-based mechanism.

## RESULTS

### Correlation between composition parameters and gene expression

*Wolbachia*’s close symbiotic relationship with its host has led to extensive genome rearrangements and the loss of many regulatory regions, as observed in other endosymbionts. Despite this genomic reduction, experimental studies have reported differential expression of *Wolbachia* genes across host developmental stages and in response to drug treatments. However, the underlying mechanisms regulating gene expression in *Wolbachia* remain largely unknown. To address this, we investigated whether patterns in nucleotide composition could provide insights into gene expression regulation in *Wolbachia*.

**Fig 1:**
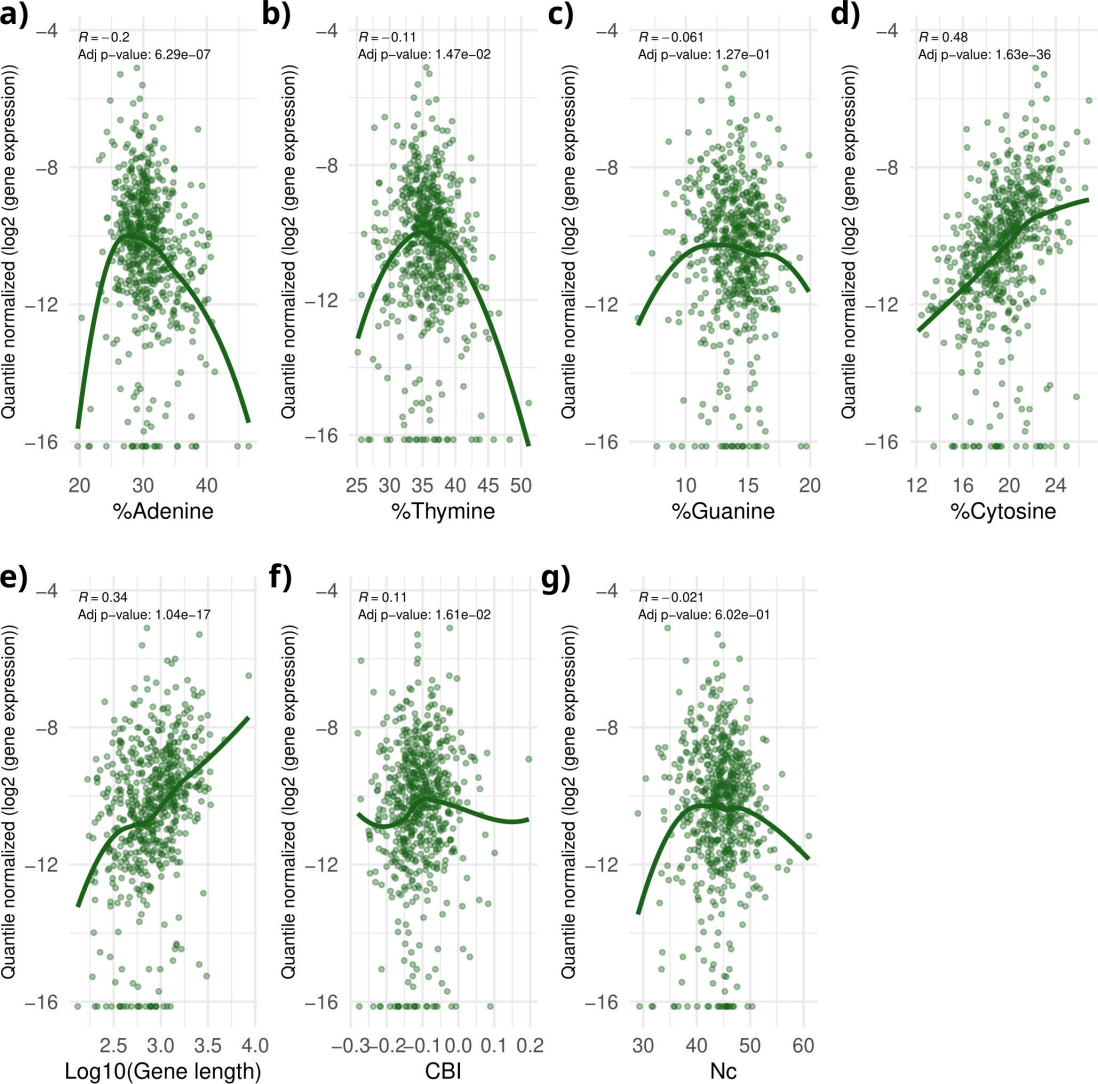
Correlation between gene composition parameters and gene expression in wOo from male host. Here, the regressions of a representative host-strain condition (*w*Oo—male) among the 55 included in the study are reported. Each graph displays individual genes as points, with gene expression shown on the *x*-axis and a gene composition parameter on the *y*-axis. Panel **a** shows %adenine, (**b**) %thymine, (**c**) %guanine, (**d**) %cytosine, (**e**) gene length (log_10_ transformed), (**f**) Codon Bias Index, and (**g**) effective number of codons. Gene expression values are quantile-normalized log_2_-transformed values. A green trend line represents the general tendency, while Spearman’s ρ and the adjusted *P*-value are displayed at the bottom of each plot. Panels d and e clearly show that there is an association between gene %C and gene length with gene expression in this host-strain condition.

**Fig 2:**
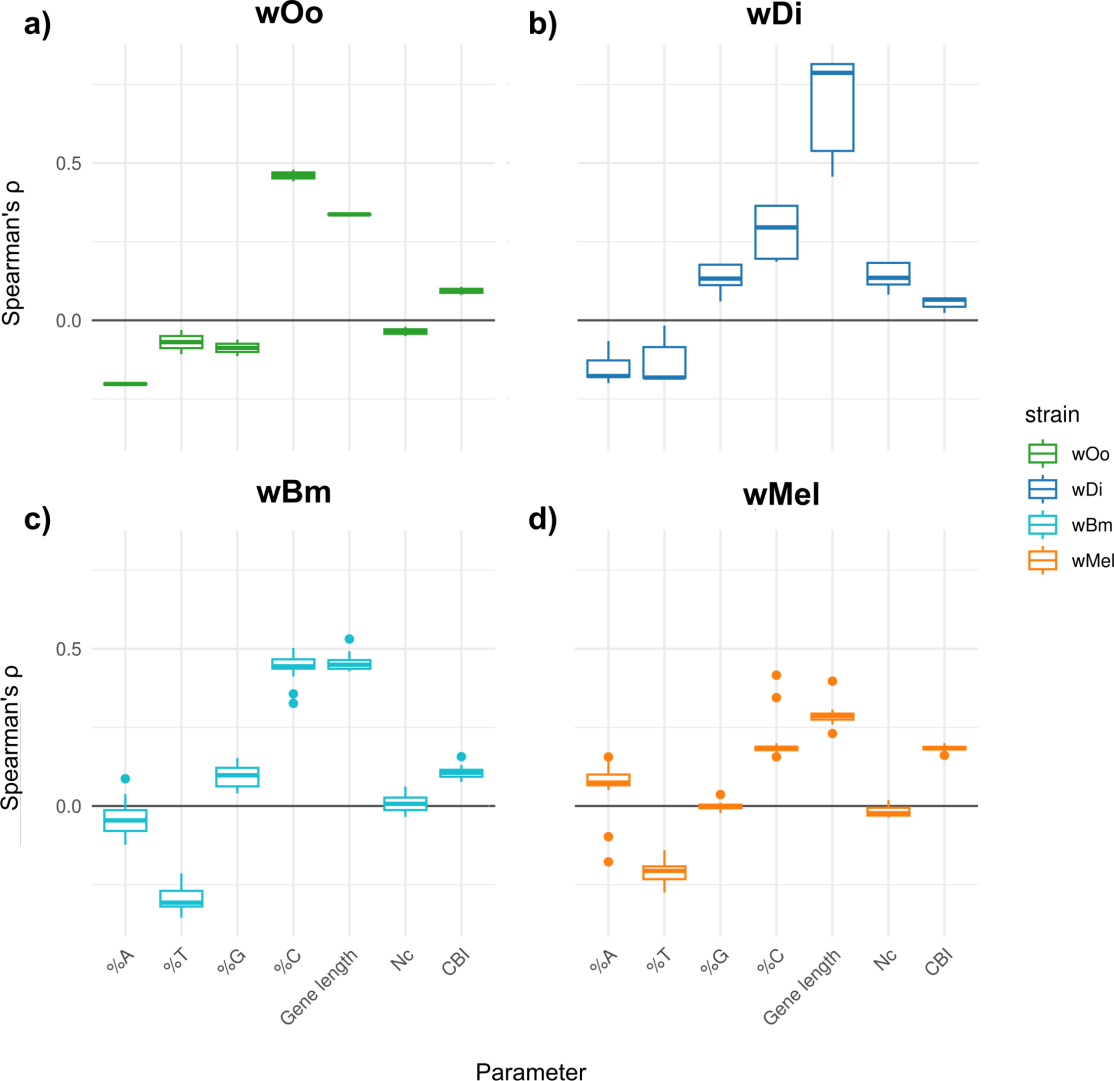
Boxplots of Spearman’s ρ values across *Wolbachia* strains. Each plot presents boxplots summarizing Spearman’s ρ values, which reflect the correlation between gene expression and various gene composition parameters. Panel **a** displays results for *w*Oo (in green), (**b**) *w*Di (in blue), (**c**) *w*Bm (in azure), and (**d**) *w*Mel (in orange). In each panel, gene composition parameters are shown on the *x*-axis, while Spearman’s ρ values are plotted on the *y*-axis. The plots demonstrate that the association between %C per gene length and gene expression is generally consistent across different host-strain conditions.

We re-analyzed RNA-seq data from 55 *Wolbachia* samples obtained from hosts at different developmental stages or after exposure to a drug, obtained from the literature (18–20, 23, 24, 33) (see Table S1 for more details about the samples). We examined the correlation between gene expression levels and various genomic features, i.e., %A, %T, %C, %G, gene length, Codon Bias Index (CBI), and effective number of codons (Nc) (as an example, Fig. 1 presents the regression plots for the *w*Oo *Wolbachia* strains from the *Onchocerca ocheng*i male sample, from the Darby et al. [18] data set). We found association in nearly all the 55 samples for %C, %T, Nc, CBI, and gene length. Indeed, significant Spearman’s correlations (*P*-values <0.05) were observed with %T in 53 out of 55 cases (96%), CBI in 50/55 (91%), %A in 31/55 (56%), %G in 20/55 (36%), and Nc in 5/55 (9%). For what concerns the sign of correlations, %C, CBI, and gene length were generally positively correlated with gene expression, whereas %T typically showed negative correlations (Fig. 2). More specifically, Spearman’s ρ values ranged as follows: for gene length from 0.23 to 0.82, %C from 0.16 to 0.50, CBI from 0.02 to 0.2, %T from −0.36 to −0.02, Nc from −0.05 to 0.19, %A from −0.21 to 0.16, and %G from −0.11 to 0.18 (Fig. 2 and Table S2).

Next, we assessed the co-correlation among nucleotide composition parameters across the four *Wolbachia* strains included in the study (*wOo*, *wDi*, *wBm*, and *wMel*). Overall, the analysis revealed largely consistent co-correlation patterns among the strains, with some notable differences (Fig. S1). For example, while %A and %C were negatively correlated in all strains, the strength of this correlation—as measured by Spearman’s ρ—varied considerably: it was strongest in *wMel* (ρ = –0.48), followed by *wBm* (–0.37) and *wOo* (–0.32), whereas in *wDi*, the correlation was much weaker (–0.03). These findings suggest that selective pressures acting on gene composition may differ among strains. Moreover, we cannot exclude the possibility that such variation could influence the relationship between gene composition parameters and gene expression.

### Experimental investigation of the DNA methylation activity of the *w*Oo MidA

One of the most expressed genes in *Wolbachia* colonizing the filarial nematode *O. ochengi* (*w*Oo) was *mid*A (18). Evidence from eukaryotes suggests that it can methylate protein arginine—an amino acid residue frequently found in nucleic acid-binding sites—which may influence the binding affinity of DNA-associated proteins (32), such as RNA polymerase. Thus, we hypothesized that MidA may influence the binding affinity of RNA polymerase (and/or other transcription-related proteins), thereby modulating gene expression based on nucleotide composition. Even if the experimental testing of this hypothesis is intriguing, it is challenging and out of the scope of this work. We decided to investigate a secondary, correlated, and more achievable hypothesis that the MidA enzyme could affect gene expression by methylating DNA itself. Generally, methylation of bacterial gene promoters is known to influence the promoter’s affinity for transcription factors, leading to changes in gene expression and epigenetic modifications (34, 35). With a few promoters expected in the endosymbiont *Wolbachia* (17), we hypothesize that intragenic DNA methylation could affect gene expression, consistent with experimental evidence showing that the efficiency of bacterial RNA polymerase is influenced by epigenetic modifications (36–39). Recently, third-generation sequencing approaches, such as SMRT PacBio sequencing, have made the investigation of DNA methylation more achievable and precise by identifying methylated nucleotides during the sequencing process. We synthesized the *wOo mid*A gene and cloned it into an expression plasmid, which was then used to transform the *Stellar E. coli* strain (*dam^−^/dcm^−^*), which lacks DNA methyltransferase activity. Then, we performed SMRT PacBio sequencing on the transformed *E. coli Stellar* (dam^−^/dcm^−^) strain after gene expression induction. We also sequenced an *E. coli Stellar* (dam^−^/dcm^−^) transformed with the empty plasmid (lacking the *midA* gene) as a negative control.

This procedure allowed us to identify 166 high-quality methylated bases: 151 on cytosine (classified as m4C-N4-methylcytosine) and 15 on adenine (classified as m6A-N6-methyladenine) (see Table S3 and Fig. S2). Investigation of the methylation pattern revealed that m4C methylations occurred in a pattern that was not well conserved, whereas m6A methylation involved a guanine-rich region at positions −4, −8, and −19 (Fig. S3). The *w*Oo *mid*A was found to be able to methylate both adenine and cytosine, without highly conserved patterns and with a 10-fold greater affinity for cytosine than adenine. This value has been determined on an *E. coli* genome containing ~50% of AT. Instead, the genome of a *Wolbachia* strain usually contains ~70% of AT, and this partially reduces the MidA affinity bias to fourfold. These findings on the DNA methylation capability of the MidA in *w*Oo are intriguing, but this result must be considered as preliminary, and further experimental validations are necessary.

### What regulates *mid*A expression?

We investigated the mechanisms that might regulate midA gene expression. To this end, we searched for the *mid*A gene across 112 *Wolbachia* genome assemblies (Table S4) and characterized the upstream regions to search for the transcription-related binding sites CtrA, Pribnow, CAAT, GC, and TATA boxes.

For one genome assembly (accession GCF_018454445.1, *Wolbachia* endosymbiont of *Rhagoletis cerasi—w*Cer5), the *mid*A gene was located on the extreme of a contig, and thus it was not possible to extract the 100 bp upstream of the gene transcription initiation site. Among the 111 regions upstream of the *mid*A gene, 104 (94%) contained perfect TATA boxes (Fig. S4), 100 (90%) perfect CtrA-binding sites (Fig. S5), and 5 (5%) contained perfect Pribnow boxes (Fig. S6).

Then we investigated the presence of CtrA-binding site and TATA boxes upstream of all the genes of the 112 *Wolbachia* genomes included in the study. This led to the discovery of a total of 2,210 CtrA-binding sites and 12,283 TATA boxes. Among all *Wolbachia* genes, only midA and formate hydrogenlyase subunit 3/multisubunit Na^+^/H^+^ antiporter resulted to have a conserved CtrA-binding site upstream (see Table S5 and Fig. S7). Instead, the TATA boxes were found with high frequency upstream of 12 genes (see Table S5 and Fig. S8), including the gene coding for the CtrA DNA-binding response regulator.

The CtrA-binding site is a sequence bounded by the CtrA response regulator protein, which is part of the two-component regulatory system Ccka/CtrA. The presence of a highly conserved CtrA-binding site upstream of the *mid*A gene locus strongly suggests that the CcKA/CtrA signal transduction pathway could have a role in the regulation of the expression of this gene. Indeed, the host could modulate the expression of the *Wolbachia mid*A gene by stimulating the Ccka receptor on the membrane of the bacterial cell. Furthermore, the presence of a conserved CtrA-binding site upstream of only two genes suggests that this could be a highly regulated mechanism subjected to a strong selective pressure. All these considerations are based on the current literature regarding the functioning of the Ccka/CtrA system, and the mechanisms still require further experimental investigation.

We also found highly conserved TATA boxes (i.e., eukaryotic regulatory sequences) upstream of the *mid*A gene. In eukaryotes, TATA boxes tend to be placed upstream of stress-responsive genes, whose expression must rapidly and variably be tuned in response to specific environmental conditions and changing physiological needs (40, 41). The TATA box is bound by a specific protein, the TATA box-binding protein (TBP), which then recruits transcription factors. *Wolbachia* does not encode TBP proteins, so the functionality of these TATA box sequences can only be hypothesized, e.g., assuming that TBPs are supplied by the host. Interestingly, we found 12 *Wolbachia* genes having highly conserved TATA boxes. These genes include *ctr*A response regulator (see above), genes that are involved in energy production and a component of the type IV secretion system.

### Is MidA a regulator of gene expression?

As stated in the "Correlation between composition parameters and gene expression" section above, *Wolbachia* gene expression globally correlates with nucleotide composition. Considering that DNA methylation is known to be involved in gene expression regulation in prokaryotes, we investigated if MidA methyltransferase activity may influence the association between gene composition parameters and gene expression. In particular, we tested if the *mid*A gene expression level correlates with an increase (or decrease) of strength of the association between nucleotide composition parameters and gene expression. We used a generalized additive mixed model (GAMM) to evaluate the association between *mid*A expression and Spearman’s *ρ* values across the 55 samples, with strain included as a random effect.

**Fig 3:**
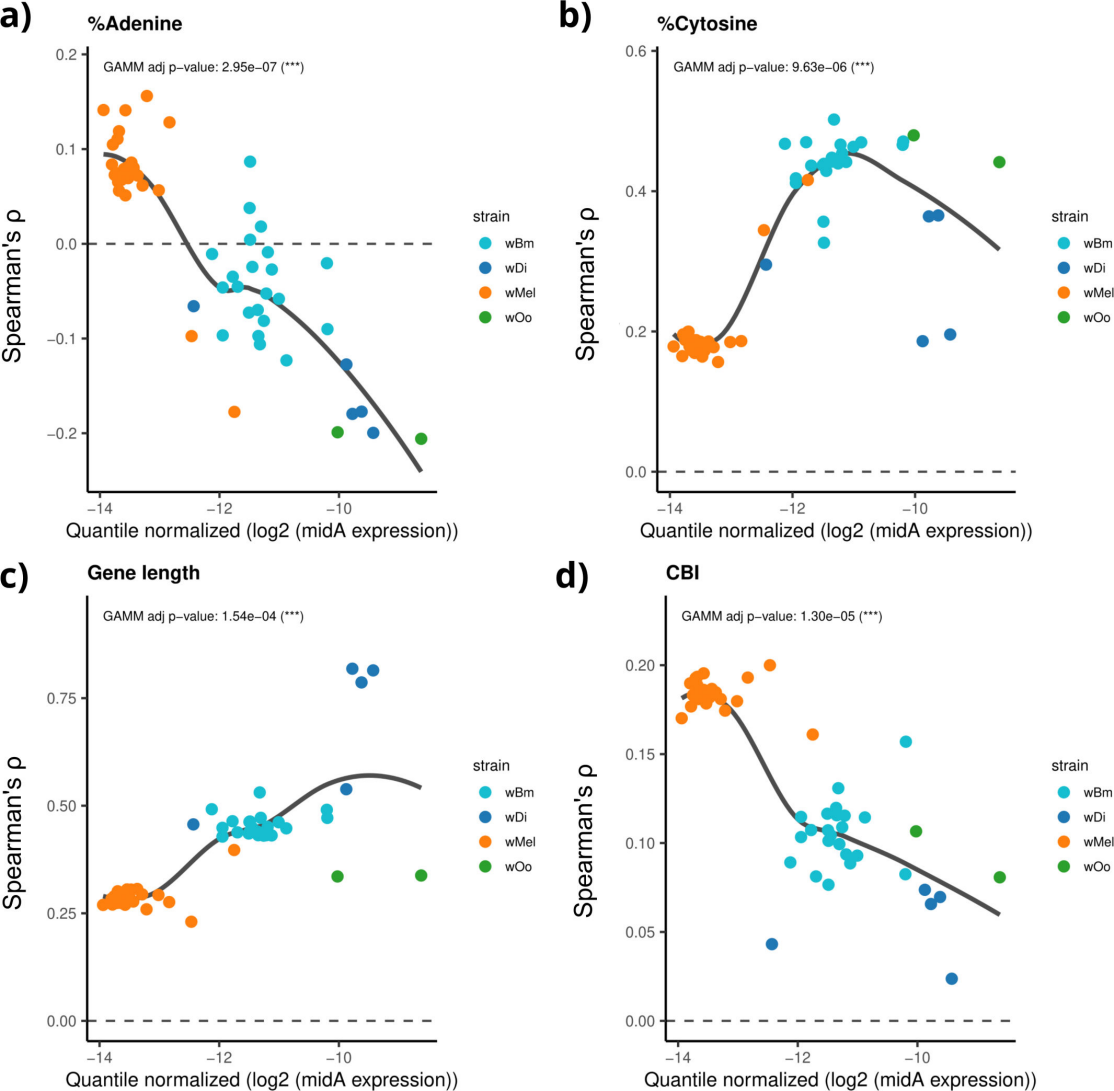
Correlation between *mid*A expression and the association of gene composition parameters with gene expression. Each scatterplot illustrates the relationship between *mid*A gene expression and the strength of association between various gene composition parameters and gene expression across the 55 samples analyzed. Each point represents a sample and is color coded by strain: azure for wBm, blue for wDi, orange for wMel, and green for wOo. The *x*-axis displays the quartile-normalized log_2_ expression of *mid*A, while the *y*-axis shows Spearman’s ρ value, indicating the correlation between a specific gene composition parameter and gene expression within that sample. The *P*-value from a generalized additive mixed model, with strain included as a random effect, is reported below each plot. Panel **a** shows %adenine, (**b**) %cytosine, (**c**) gene length, and (**d**) effective number of codons. The plots indicate that variations in *mid*A expression are associated with different correlations between gene nucleotide composition and gene expression. For example, in host-strain conditions where MidA is highly expressed, there is a positive correlation between %A and gene expression (Spearman’s ρ > 0), whereas when MidA is downregulated, this correlation turns negative. Thus, genes with a high %A tend to be downregulated when *mid*A is highly expressed, and conversely, they are upregulated when *mid*A expression is low.

The increase of the *mid*A gene expression resulted to be significantly associated with the increase of Spearman’s *ρ* values for %C, %G, %T, Nc, and gene length (Fig. 3; Fig. S9 to S11) and with decrease for %A and CBI (Fig. 3). The most evident correlation shift was observed for %A, being positive when the *mid*A gene is poorly expressed and gradually decreasing, becoming negative in samples with highly expressed *mid*A. The *mid*A gene expression was also associated with a reduced influence of Nc and CBI on gene expression, suggesting a diminished role of codon composition in affecting gene expression. Interestingly, *mid*A expression correlates with an increased effect of gene length on gene expression, coherently with the idea that the MidA methyltransferase acts directly on the gene sequence, possibly through mechanisms such as methylation (i.e., the longer the gene, the greater the effect).

Then, we evaluated if other genes presented a gene expression pattern similar to the *mid*A gene across the 55 samples. The principal component analysis (PCA) among the expression patterns of the single-copy core genes shared among *w*Oo, *w*Di, *w*Bm, and *w*Mel showed that no other gene has an expression pattern overlapping that of the *mid*A gene. Indeed, the highest correlation value was 0.84, the lowest −0.87, and the median value was 0.02 (Fig. S12). In general, the absence of clusters of co-expressed genes is also evident from PCA analysis (Fig. S13).

### What could MidA regulate?

As stated above, the expression of the *mid*A gene could be associated with the downregulation of %A rich genes and upregulation of %A poor genes. Coming gene nucleotide composition and Clusters of Orthologous Groups (COG) annotation, we found a total of 63 low %A genes and 41 high %A genes.

**Fig 4:**
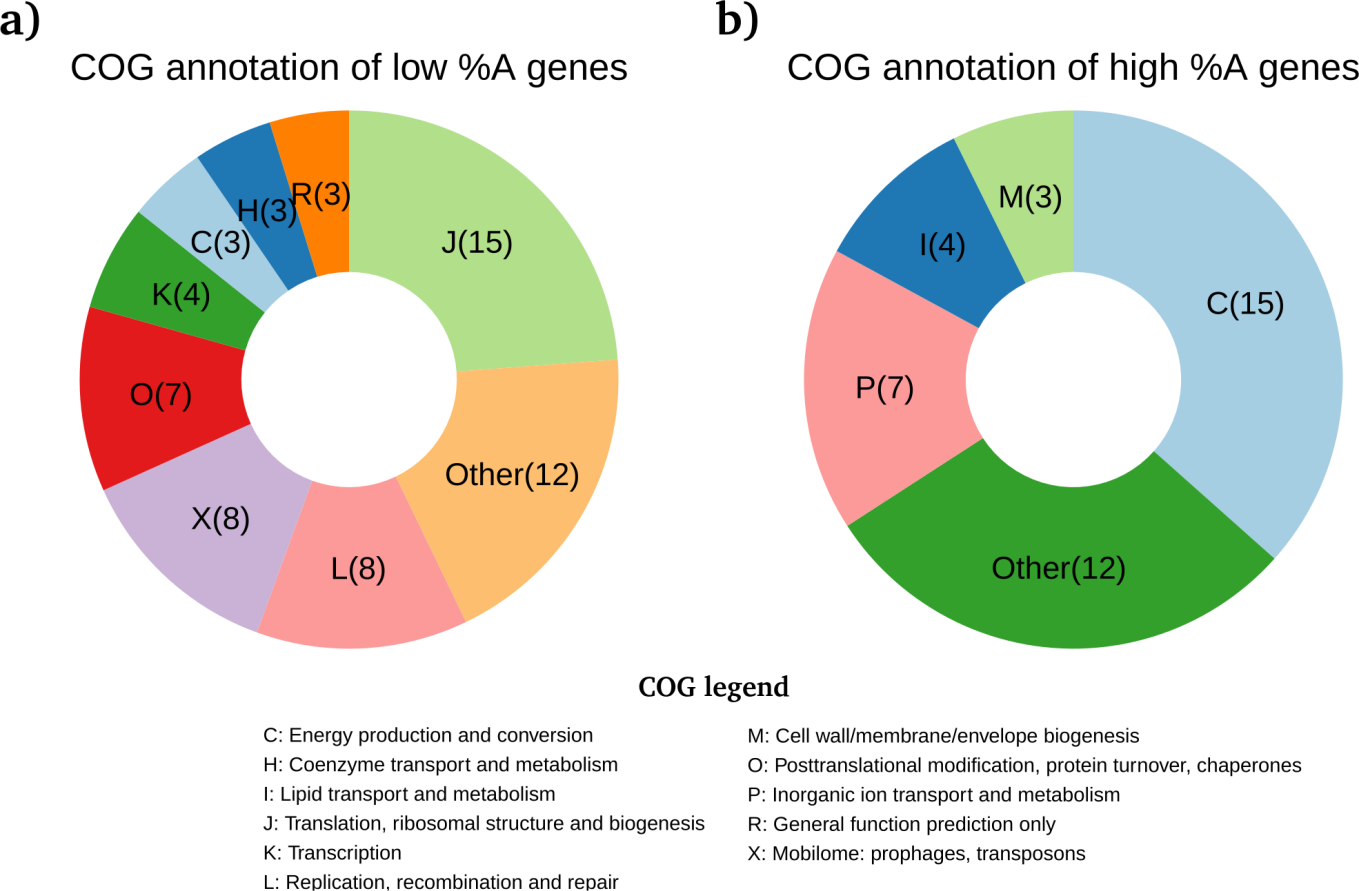
COG annotation of genes with the highest and lowest %A content in *w*Oo, *w*Di, *w*Bm, and *w*Mel. The pie charts display the distribution of Clusters of Orthologous Group annotations among the genes with the highest and lowest adenine content (%A) in at least one of the *w*Oo, *w*Di, *w*Bm, or *w*Mel *Wolbachia* genomes. Panel **a** (left) shows genes with low %A, while panel **b** (right) shows genes with high %A. COG functional categories are represented by letters and color coded in each chart. The legend for the COG letters is shown below the figure. Several genes with high %A content are involved in energy production, while %A-poor genes are associated with translation. Therefore, differential expression of *mid*A—which appears to modulate gene expression based on %A content—could influence *Wolbachia* metabolism by shifting the balance between these two metabolic pathways.

Among the low %A genes, the most frequent COG categories were “translation, ribosomal structure, and biogenesis” (J) with 15/63 (24%). Conversely, among the high %A genes, the most frequent COG categories were “energy production and conversion” (C) with 15/41 (37%) (Fig. 4; Fig. S14). This suggests that the expression of the *mid*A gene, possibly induced by the Ccka/CtrA system, could shift the *Wolbachia*’s metabolism from energy production oriented to gene translation oriented. Coherently, when *mid*A expression is low, T-rich genes are also poorly expressed, thereby reducing ATP consumption due to RNA synthesis.

### MidA conservation across *Wolbachia* genus

Once we found evidence coherent with the hypothesis that MidA methyltransferase could affect gene expression in *Wolbachia*, we investigated more in depth its conservation and evolution. More in detail, we compared the MidA protein phylogeny to the *Wolbachia* phylogeny to assess if the gene is frequently horizontally transferred and its conservation among the four strains studied (*w*Oo, *w*Di, *w*Bm, and *w*Mel).

The orthology analysis on 112 *Wolbachia* genome assemblies identified a total of 3,561 orthologous groups, including 263 single-copy core genes. None of the single-copy core genes were recombined (on the basis of the PHI parameter, see Materials and Methods). After the removal of *mid*A and the trimming of gene alignments, the obtained concatenate had a length of 2,219,973 bp, and species tree was obtained.

A way to assess the level of horizontal gene transfer (HGT) of the MidA protein is to compare its phylogenetic tree with the *Wolbachia* species tree. Although this approach is widely used, it carries a significant bias: it is reasonable that short genes often contain less phylogenetic information than longer ones, which can lead to phylogenetic trees that are more discordant with the species tree (which is inferred from multiple concatenated gene sequences). The OrthoFinder pipeline, used for the orthologs analysis, includes a step in which the phylogeny of each core gene is computed and stored. Thus, to assess the level of horizontal gene transfer in *MidA*, we calculated the topological distance between the species tree and the tree of each of the 263 single-copy core genes. Then, we compared MidA tree’s distance to those of the other genes, taking gene length into account. As shown in Fig. S15, tree distance decreases with increasing gene length. The distance of *MidA* is comparable to that of other single-copy core genes of similar length, including ribosomal proteins, which are well known to be generally unaffected by horizontal gene transfer.

**Fig 5:**
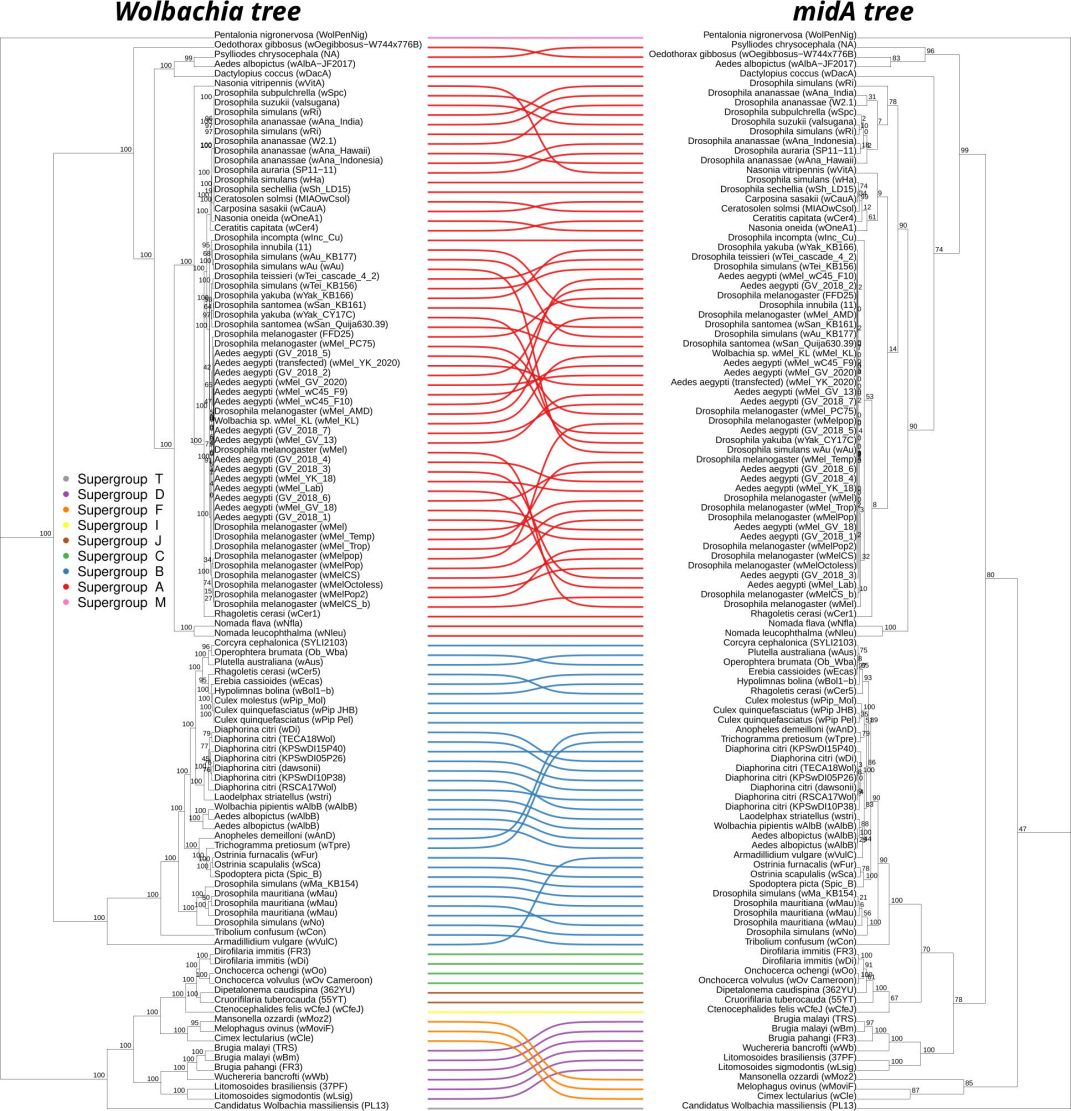
Comparison of *Wolbachia pipientis* vs *mid*A phylogenetic trees. The ML trees obtained from: on the left, the nucleotide concatenate of the single-copy core genes of 112 representative *Wolbachia* genomes; on the right, the nucleotide sequences of the 112 *mid*A gene retrieved from the same genomic data set. Colored lines connect the corresponding strains on the trees, and bootstrap support values are reported in the trees. Lines are colored on the basis of the strain supergroup, following the legend placed on the left. As shown by the colored lines, the supergroups are mainly maintained among the two trees, suggesting a general conservation of the topologies. This is coherent with a main vertical transmission of the *mid*A gene and few horizontal gene transfer events (as also confirmed by the reconciliation analysis).

Furthermore, we generated a bootstrapped maximum likelihood (ML) phylogenetic tree using MidA protein sequences and visually compared it to the species tree (Fig. 5). Then, we subjected the two trees to reconciliation analysis, a method useful for the identification of HGT. The two topologies were mainly congruent, and the reconciliation analysis identified five putative HGT events, involving three strains belonging to supergroup A (*w*Oegibbosus-W744 × 776B—accession GCF_936270145.1, *w*Yak_KB166—GCF_018467115.1, and *w*Mel—GCF_016584425.1), one to B (*w*stri—accession GCF_007115015.1), and one to the supergroup F (*w*Moz2—accession GCF_020278625.1).

The alignment of MidA protein sequences from *w*Oo, *w*Di, *w*Bm, and *w*Mel reveals a high degree of conservation, except for the central region, which is more variable (Fig. S16). Notably, the putative DNA-binding regions, marked by red asterisks in Fig. S16, display strong conservation. This suggests that the MidA proteins from *w*Di, *w*Bm, and *w*Mel may possess methylation activity comparable to that of *w*Oo.

Overall, these results show that MidA methyltransferase is highly conserved across the *Wolbachia* phylogeny, with no evidence of gene duplications and only a few HGT events, consistent with the hypothesis that this gene plays a pivotal role in the bacterium’s metabolism.

## DISCUSSION

*Wolbachia* is one of the most widespread endosymbiotic bacteria (11), with an important impact on the survival and/or reproduction of several arthropod and filarial nematode host species (2, 5, 42). The intracellular symbiotic/parasitic lifestyle led the bacterium to lose several regulatory regions (17). Despite experimental evidence suggesting the existence of a coordination between *Wolbachia* gene expression and host physiology (e.g., developmental stages), the mechanisms underlying this phenomenon remain to be clarified.

The re-analysis of the RNA-seq data from 55 samples of 4 *Wolbachia* strains led us to propose that gene nucleotide composition and DNA methylation can have a role in the regulation of *Wolbachia* gene expression. Moreover, our analyses suggest a possible role for the MidA methyltransferase and the CcKA/CtrA signal transduction system.

**Fig 6:**
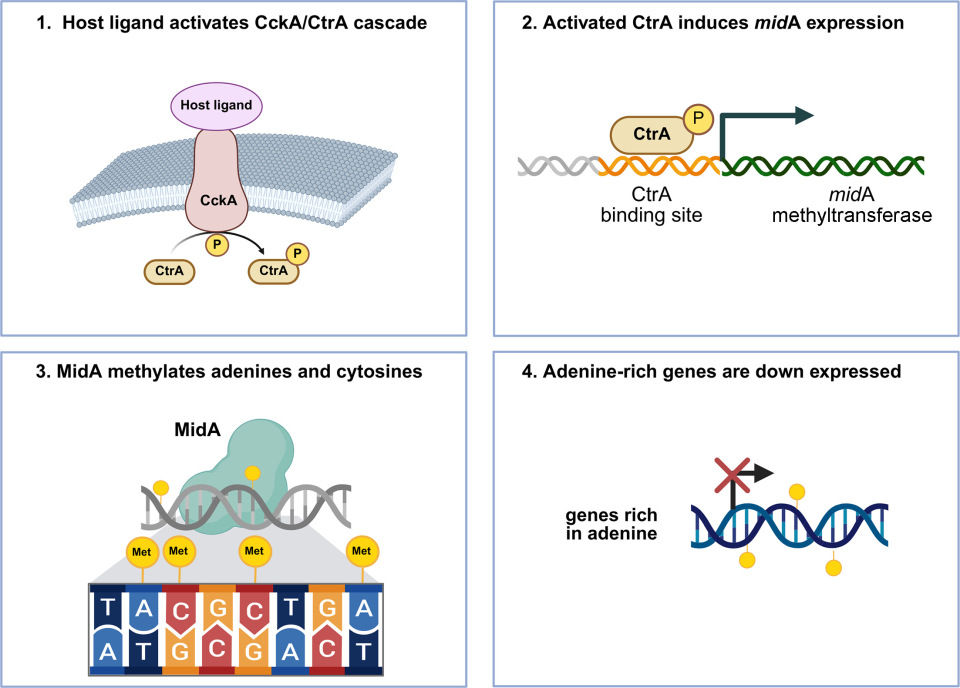
Proposed model of MidA-mediated gene regulation via the CckA/CtrA signaling cascade in *Wolbachia*. A host-derived ligand activates the CckA/CtrA two-component system (step 1), resulting in phosphorylation and activation of CtrA. Activated CtrA binds to the promoter region of *mid*A, inducing its expression (step 2). The MidA methyltransferase subsequently methylates adenines and cytosines in the genome (step 3), leading to transcriptional repression of adenine-rich genes (step 4). This model suggests a possible host-responsive epigenetic mechanism by which *Wolbachia* modulates gene expression.

Here, we propose a model for the host-mediated gene expression regulation in *Wolbachia,* graphically summarized in Fig. 6. The model is composed of five steps: (i) specific molecules in the host cells activate the CckA membrane histidine kinase; (ii) CckA phosphorylates the intracellular CtrA response regulator; (iii) the phosphorylated CtrA binds to the CtrA-binding site upstream of the *mid*A gene, inducing its expression in *Wolbachia*; (iv) the MidA enzyme methylates adenine and cytosine of the *Wolbachia* genome (and possibly proteins involved in the transcription); (v) the expression of several *Wolbachia* genes changes on the basis of their nucleotide content, particularly %A-rich genes are downexpressed. Moreover, the presence of a conserved TATA box upstream of the *ctr*A gene could suggest a possible role for the host in the regulation of the *ctr*A gene expression.

While the hypothesized mechanism may not lead to highly precise regulation of gene expression, it could be a rough yet functional mechanism, sufficient for the establishment of a successful intracellular symbiosis. In the future, it would be interesting to investigate whether similar mechanisms are present in other endosymbiont or intracellular organisms.

Despite the results presented in this work are far from being definitive, we hope to have opened a new way in understanding the symbiotic relationship between *Wolbachia* and its hosts. In our opinion, this is a magnificent example of how evolution works, recycling metabolic pieces for similar aims, sometimes reaching surprisingly stable equilibria.

## MATERIALS AND METHODS

### Data set reconstruction and data normalization

Table S2 and gff map files of the supplementary information of Chung et al. were retrieved (33). The table contains the gene expression quantifications computed by Chung and colleagues (33), re-analyzing 7 RNA-seq studies on *Wolbachia* (18, 20–24), for a total of 128 replicates from 62 host conditions (i.e., developmental stages or exposure to doxycycline). More in detail: (i) 2 replicates for each of 2 samples of *O. ochengi* (*Wolbachia* strain *w*Oo), 1 from whole male and 1 from female gonads, from reference (18); (ii) 1 replicate for each of 5 host developmental stages of *Dirofilaria immitis* (*Wolbachia* strain *w*Di) from reference (19); (iii) 3 replicates for the *Aedes albopictus* cell line infected with wMel strain, before and after exposure to doxycycline (*Wolbachia* strain *w*Mel), from reference (20); (iv) 12 total replicates representing 7 developmental stages of *D. immitis* (*Wolbachia* strain *w*Di), from (21); (v) 55 replicates representing 24 developmental stages of *Drosophila melanogaster* (*Wolbachia* strain *w*Mel), from reference (22); (vi) 2 replicates for each of 7 developmental stages of *Brugia malayi* (*Wolbachia* strain *w*Bm), from reference (23); (vii) 2 replicates for each of 15 developmental stages of *B. malayi* (*Wolbachia* strain *w*Bm), from reference (24). Preliminarily, we excluded from the data set all the samples from reference (21) because only 8% of the gene expression quantifications were greater than 0. The obtained data set accounts for a total of 55 samples from 4 *Wolbachia* strains: 2 for *w*Oo, 5 for *w*Di, 22 for *w*Bm, and 26 for *w*Mel (or *w*MelPop), for details see Table S1. The analyses were performed using R (https://www.R-project.org/).

Gene expression values were normalized to make them comparable among studies and conditions. For each condition in each study, the gene expression values were expressed as


$$Log_{2}expr=\sum s[\sum log_{2}(1+expr_{s,i}/\sum 1+expr_{s,i})]/S_{num}$$

where

*s* = sample (or replicate); *i* = gene; expr_*s*,*i*_ = the gene expression value of gene *i* in sample (replicate) *s*; *S*_num_ = total number of samples. This normalization formula derives from the log_2_ fold change (FC) formula often used to compare two conditions in RNA-seq studies:


$$Log_{2}FC=Log_{2}expr_{condition1}-Log_{2}expr_{conditio2}=\sum s[\sum i\;log_{2}(expr_{s,i}/\sum i\;expr_{s,i})]/S_{condition1}-\sum s[\sum i\;log_{2}(expr_{s,i}/\sum \;iexpr_{s,i})]/S_{condition2}$$

Log_2_FC allows us to compare values from different conditions in the same experiment. To make the normalized values more comparable among the studies, they were converted into quantiles. Indeed, the quantiles may be less biased by sample-specific features, such as the number of genes.

### Genes composition parameters determination

The genome assemblies of *w*Bm (AE017321.1), *w*Di (www.nematodes.org: *w*Di 2.2), *w*Oo (HE660029.1), and *w*Mel (AE017196.1) were retrieved and passed to Prodigal (43) for open reading frame calling. For each ORF, Codon Bias Index (which measures how much a gene uses a subset of optimal codons, ranging from 0 for random usage to 1 for maximum bias), the effective number of codons, and the ORF length were computed using CodonW tool (44), while %A, %T, %G, and %C were computed using an in-house Perl script. Conventionally, ORF sequences correspond to the transcribed messenger RNA sequence, which is complementary to the DNA template strand. Thus, the nucleotide composition of the genes is derived by complementing the ORF compositions: the %A of a gene is determined as the %T of the corresponding ORF, and so on. All subsequent analyses will focus on the nucleotide composition of the genes rather than on ORF compositions.

The co-correlation among the gene’s composition parameters was computed separately for each strain using Spearman’s correlation test.

### Correlation between gene composition parameters and gene expression

The relationship between each gene composition parameter (CBI, Nc, length, %A, %T, %G, and %C) and gene expression was investigated. More in detail, for each host-strain condition, Spearman’s correlation test was used to study the relationship between the gene expression and each of the gene composition parameters. Separately for each *Wolbachia* strain, the *P*-values were then adjusted for multiple comparisons using the Bonferroni post hoc correction. For each analysis, Spearman’s ρ and the *P*-values were obtained. Spearman’s ρ indicates the strength and direction of change of gene expression in relation to the gene composition parameter: the higher the absolute value of Spearman’s ρ, the more the gene expression and gene composition parameter are correlated. Positive values indicate a positive association, while negative Spearman’s ρ indicates the opposite.

### Experimental investigation of the DNA methylation activity of the *w*Oo MidA methyltransferase

The *w*Oo SAM-dependent *mid*A gene was artificially synthesized with codon usage optimized (performed by Eurofins Scientific company) and cloned into the XhoI and SalI sites of the pCOLD III expression vector (Takara Bio). The recombinant construct was transformed into *Escherichia coli* Stellar Competent Cells (*dam*^–^/*dcm*^–^) (Takara Bio) according to the supplier’s protocol (ClonTech; Protocol-at-a-Glance, PT5056-2). The *E. coli* strains were cultured in LB broth medium supplemented with ampicillin. Before inducing expression, PCR with custom primers targeting the gene of interest (primer forward: CACAAAGTGCATATGGAGCT; primer reverse: AGCAGAGATTACCTATCTAGA) was performed on the extracted bacterial DNA to verify the outcome of transformation. The expression of the *mid*A gene was then induced at 15°C, according to the protocol provided by the manufacturer for the Cold Shock Expression System of pCold plasmids (Takara Bio). Twenty-four hours after the induction, the expression of the *w*Oo *mid*A gene was verified by SDS-PAGE. DNA was then extracted using the NucleoSpin Microbial DNA Mini Kit (Macherey Nagel) and subsequently subjected to long reads sequencing using Sequel I (Pacific Biosciences), after quality check using Qubit Fluorometer (dsDNA High Sensitivity) and Agilent 2200 TapeStation (Agilent Technologies, Santa Clara, CA). Assembly of reads was done using the Microbial Assembly pipeline provided by SMRT Link v.10.1 with a minimum seed coverage of 30×. The assembled genome was used as a reference in the downstream analysis. The methylation status of each base of the obtained reads was then determined using the “Base Modification Analysis” pipeline included in SMRT-Portal, based on the pbalign v0.3.1 and ipdSummary v2.3 tools. The pipeline performs base modification and modified base motifs detection. A negative control experiment was carried out transforming *E. coli* Stellar Competent Cells with a pCOLD III vector without the *w*Oo *mid*A gene inserted. A similar approach has been used in other studies in literature (45, 46).

The 95th percentile of mean quality value (QV) and coverage values obtained from the control experiment was set as minimum thresholds for the identification of methylated bases on the transformed *E. coli* strain. These last analyses were performed using R.

### Analysis of the region upstream of the midA gene

The positions and orientation of the *mid*A genes on the 112 genome assemblies were determined by BLASTn searches. Then, for each genome assembly, the 100 nucleotides upstream of the *mid*A gene were extracted and screened for the presence of some of the most important regulatory regions: (i) CtrA-binding site (motif TTAA-N7-TTAA [47]); (ii) Pribnow box (motif TATAAT [48]); (iii) CAAT box (motif HYYRRCCAWWSR [49]); (iv) GC box (motif WRDRGGHRKDKYYK [49]); (v) TATA box (motif TATAWAWR [49]). Perfectly matching motifs and one-mismatched motifs were considered for further analyses. The presence of CckA and CtrA in the 112 *Wolbachia* genomes was then evaluated by BlastP search, using as reference the CckA and CtrA sequences already reported in reference (28). Finally, the positions of the canonical regulatory regions upstream of the *mid*A gene and the presence/absence of CckA/CtrA in the 112 *Wolbachia* genomes were visualized using the gplots R library (50).

### Analysis of the regions upstream of all genes in the 112 *Wolbachia* genomes

On the basis of previous results on the *mid*A gene, TATA boxes were searched between positions −20 and −70 upstream of all the genes of the 112 *Wolbachia* genomes, and CrtA-binding sites between the positions 0 and −30. The presence of short AT-rich sequences, like TATA box and CtrA-binding site, in AT-rich genomes (such as *Wolbachia*) could be due to chance. The possible functionality of these sequences has been assessed by investigating whether TATA or CtrA-binding sites are enriched upstream of specific genes in the *Wolbachia* genomes. More in detail, for each of the ortholog genes previously identified using OrthoFinder (see above), the frequency of *Wolbachia* strains having upstream TATA or CtrA-binding site was investigated using R. Lastly, both for TATA box and CtrA-binding site, the genes present in at least 100 out of 112 *Wolbachia* genomes and having the regulatory box in >80% of the genomes were retrieved and annotated using the Clusters of Orthologous Groups database.

### Correlation between *mid*A transcription and the association between gene composition parameters and expression

To examine whether MidA activity influences the relationship between gene composition parameters and gene expression, a GAMM was applied using the mgcv R library (https://CRAN.R-project.org/package=mgcv). Specifically, the association between *mid*A gene expression and Spearman’s ρ values for each gene composition parameter was assessed, using *Wolbachia* strain as a random effect. Resulting *P*-values were adjusted using the Benjamini-Hochberg procedure to control for multiple testing.

### Principal component analysis of gene expression

The presence of other genes with expression patterns comparable to that of *mid*A gene was tested as follows. Exploiting the gene orthologous information included in the Table S6 of Chung et al. 2020 (33), we performed a Spearman co-correlation among the expressions of the 546 single-copy core genes shared among *w*Oo, *w*Di, *w*Bm, and *w*Mel. The expression patterns were also investigated by principal component analysis, using R.

### Analysis of the genes with high and low percentage of adenine

The expression of the genes with higher or lower %A was found to be more affected by the action of the MidA methyltransferase. To investigate the effects of this mechanism on the *Wolbachia* metabolism and physiology, genes with low or high %A values were studied as follows. A data set including 112 high-quality *Wolbachia* genome assemblies spanning the host genetic diversity was reconstructed, including 111 assemblies retrieved from the Genome Taxonomy Database (GTDB) and the wDi genome used for the previous analysis (indeed, the wBm, *w*Mel, and *w*Oo assemblies were already present in the GTDB database) (see Table S4). The genome assemblies were subjected to Prodigal (43) for ORF calling. All the amino acid sequences from the 112 *Wolbachia* strains included in the study were annotated on the basis of the Clusters of Orthologous Genes database. For each strain, among the COG-annotated genes, the 10 with lower %A (“low %A genes”) and the 10 with higher %A (“high %A genes”) were retrieved. The pattern of presence/absence and %A value of all these retrieved genes was graphically investigated by producing a heatmap using the gplots R library.

### Conservation of midA sequence across *w*Oo, *w*Di, *w*Bm, and *w*Mel

The MidA amino acid sequence from *w*Oo, *w*Di, *w*Bm, and *w*Mel was retrieved from the ORF obtained, aligned using MUSCLE 3.8.31, and visualized using the Color Align Conservation online tool (51). DNA-binding sites were inferred on the DP-Bind web server (52).

### Comparison between species tree and MidA tree

The amino acid sequences from the 112 *Wolbachia* genomes data set (see above) were passed to OrthoFinder (53) for ortholog analysis. The nucleotide sequences of the single-copy core genes were then retrieved, aligned using the MUSCLE 3.8.31 tool (54), tested for recombinations using the PHI index (1,000 permutations) using the PhiPack tool (55), trimmed using the trimal tool (-gt 0.5 setting) (56), and finally concatenated, after removing the MidA ortholog group. The obtained concatenate was passed to RAxML8 (57) for phylogenetic analysis using the GTR + I + G model, as previously determined using ModelTest-NG tool (58).

The OrthoFinder pipeline includes a step in which the phylogeny of core genes is computed. We compared the topology of each of these phylogenetic trees to the concatenated gene tree (i.e., the *Wolbachia* species tree) by Penny and Hendy distance metrics (59), using the dist.topo function of the R library Ape (60). For each single core gene, the mean gene length was computed using the R library Ape. Then, gene lengths and topology distances were visualized on a scatter plot.

Lastly, the MidA ortholog group was subjected to phylogenetic analysis following the same flow described above (using the TVM + I + G model). The obtained trees were then compared using the Cophylo R library (61) and subjected to reconciliation analysis to determine horizontal gene transfer events, using the GeneRax tool (62).

## AFTER EPUB

*[This article was published on 15 August 2025 with errors in Acknowledgments. The Acknowledgments were corrected in the current version, posted on 9 September 2025.]*

## SUPPLEMENTAL MATERIAL

Supplemental figures msystems.00779-25-s0001.pdf Figures S1 to S16.

Table S1 msystems.00779-25-s0002.xlsx Information about the samples included in the analyses.

Table S2 msystems.00779-25-s0003.xlsx Sample MTase percentiles and statistics.

Table S3 msystems.00779-25-s0004.xlsx Methylated motifs identified using SMRT PacBio sequencing.

Table S4 msystems.00779-25-s0005.xlsx Information about the *Wolbachia* strains included in the analyses.

Table S5 msystems.00779-25-s0006.xlsx Genes presenting highly conserved CtrA or TATA box regulatory regions upstream in the 112 *Wolbachia* genomes.

ASM does not own the copyrights to Supplemental Material that may be linked to, or accessed through, an article. The authors have granted ASM a non-exclusive, world-wide license to publish the Supplemental Material files. Please contact the corresponding author directly for reuse.
