# Supplementary material for: Nucleotide composition shapes gene expression in Wolbachia pipientis: a role for MidA methyltransferase?
Source: mSystems. 2025 Aug 15;10(9):e00779-25. doi: 10.1128/msystems.00779-25 (PMC12455994; doi:10.1128/msystems.00779-25)
Supplement: Supplemental figures — Figures S1 to S16. [file msystems.00779-25-s0001.pdf]

Supplemental material

Nucleotide Composition Shapes Gene Expression in *Wolbachia pipientis*: A Role for MidA methyltransferase?

Stella Papaleo[1$], Simona Panelli[1], Ibrahim Bitar[2], Lodovico Sterzi[1], Riccardo Nodari[1,3], Francesco Comandatore[1*$]

1. Department of Biomedical and Clinical Sciences, Pediatric Clinical Research Center "Romeo and Enrica Invernizzi", University of Milan, 20157 Milan, Italy.
2. Biomedical Center, Faculty of Medicine, Charles University, Pilsen, Czechia.
3. Istituto Nazionale di Genetica Molecolare (INGM) "Romeo and Enrica Invernizzi", University of Milan, 20157 Milan, Italy.

* Corresponding author

$ The authors contributed equally

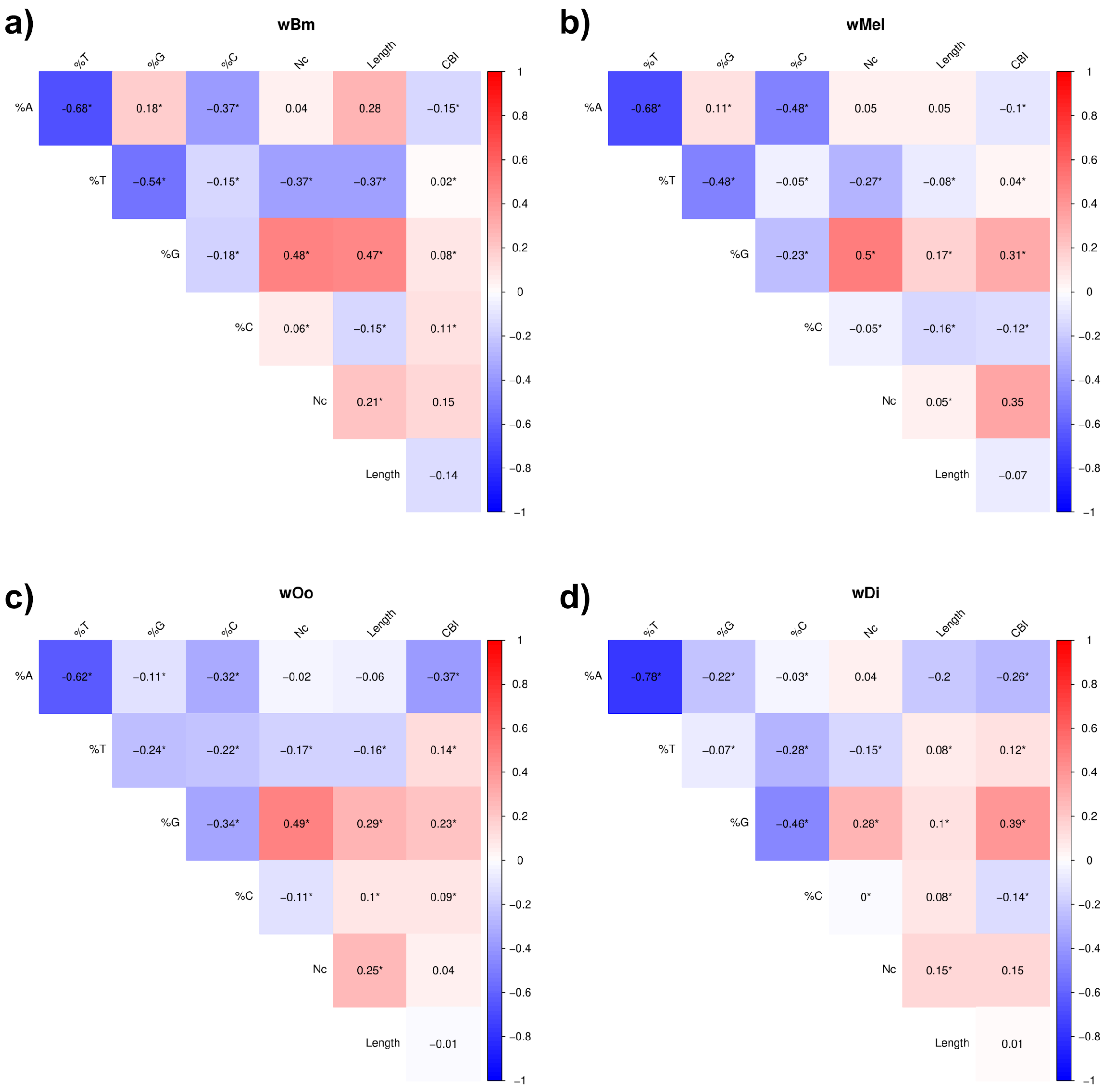

Figure 1: Co-correlation heatmaps of genomic features across four *Wolbachia* strains

Each panel (a–d) shows the Spearman's *ρ* coefficients between genomic variables including nucleotide composition (%A, %T, %G, %C), nucleotide diversity (Nc), gene length, and codon bias index (CBI) for the *Wolbachia* strains: (a) *wBm*, (b) *wMel*, (c) *wOo*, and (d) *wDi*. Positive correlations are shown in red, and negative correlations in blue, with intensity indicating the strength of the correlation. Asterisks (*) denote statistically significant correlations (p < 0.05). The analysis reveals both shared and strain-specific patterns in genomic feature relationships.

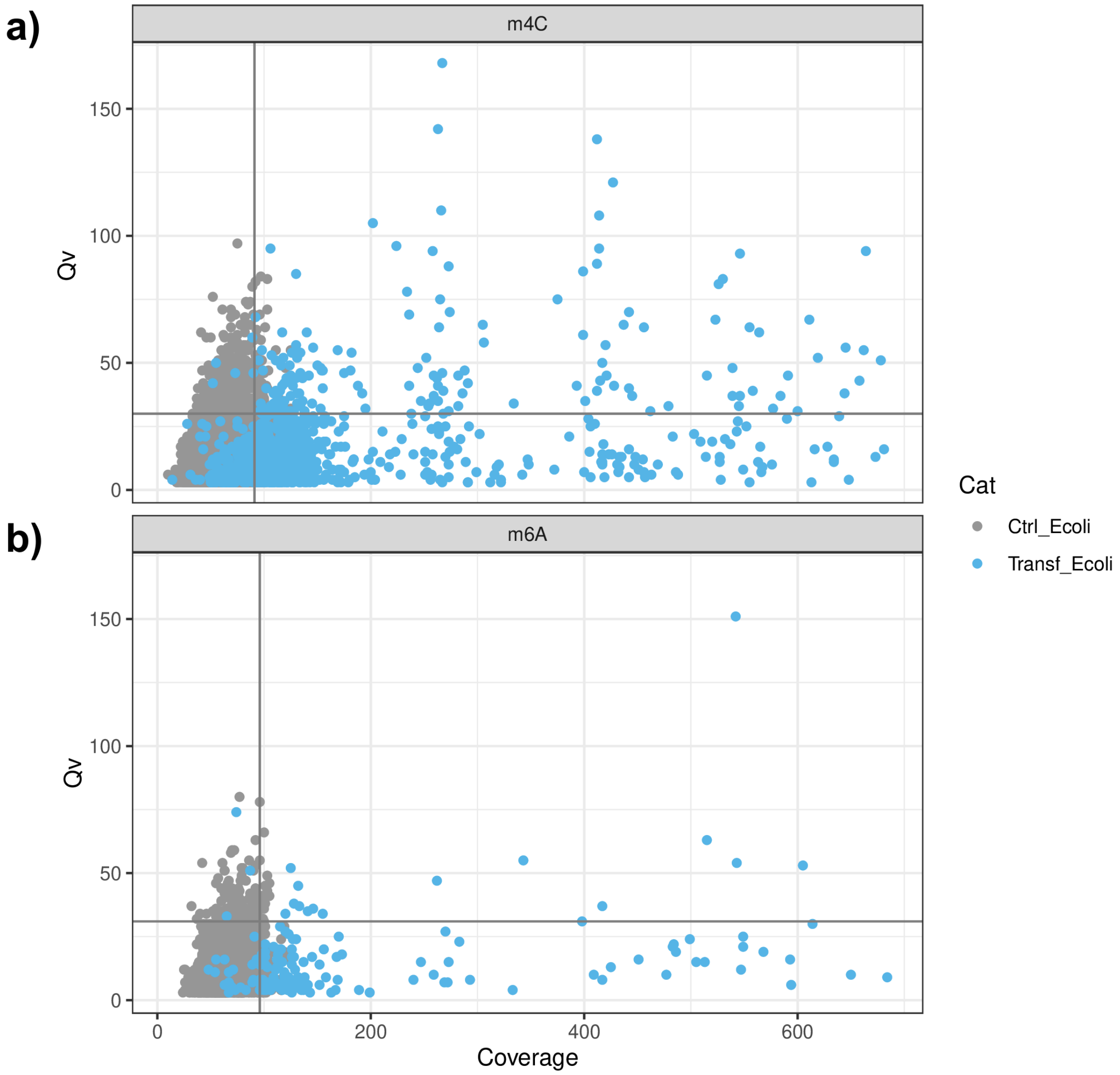

Figure S2. Scatter plot of Qv and Coverage of methylated motifs

The Qv and coverage values of all the putative methylated motifs obtained from the control and the transformed Stellar *Escherichia coli* (dam-/dcm-) strain were studied to determine the threshold for the calling of methylated positions. Azure points refers to methylation sites on the transformed *E. coli* strain, while gray on the control strain. Horizontal and vertical lines identify respectively the 95 percentile of Qv and coverage values for the control *E. coli* strain. The Qv vs coverage scatter plot relative to m4C methylations of cytosines is reported in plot (a), for m6A methylation of adenines in plot (b).

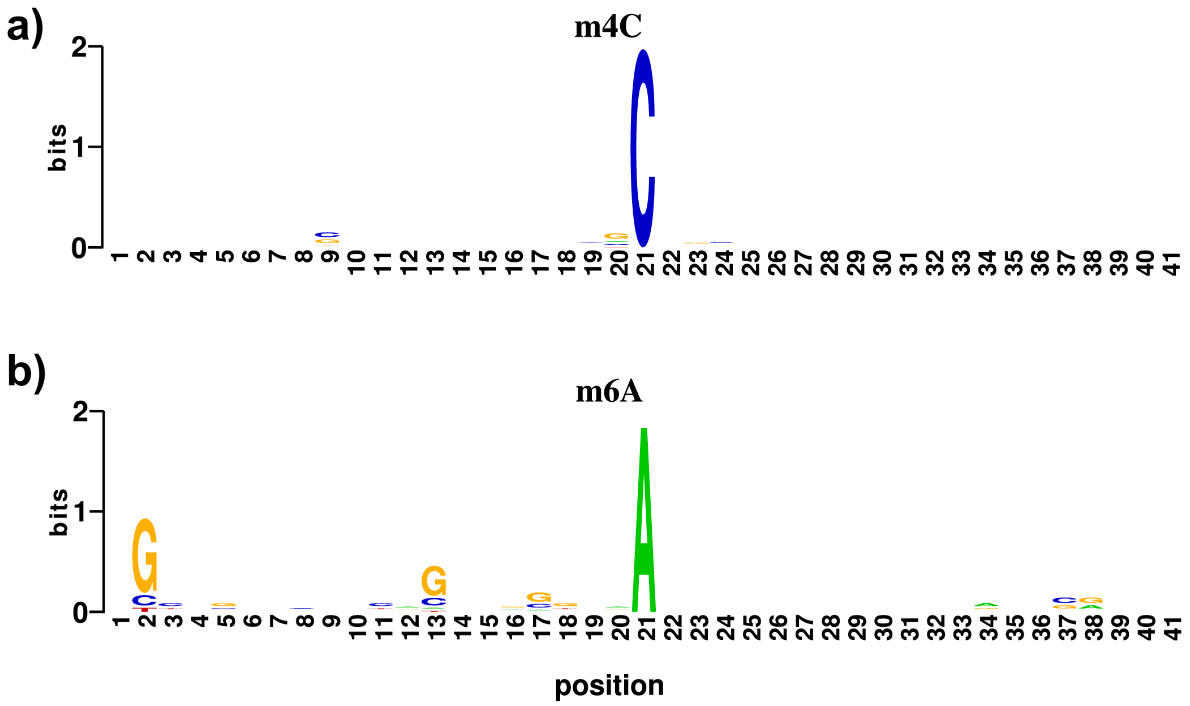

Figure S3. Methylation patterns of MidA of *Wolbachia* endosymbiont of *Onchocerca ochengi* (*w*Oo)

Sequence logo of the sequences flanking the methylated position (position 21, in the middle) found in the genome of Stellar *Escherichia coli* dam-/dcm- strain expressing the *mid*A gene of *Wolbachia* endosymbiont of Onchocerca ochengi (*w*Oo). The height of the letters depends on the conservation of the positions flanking the methylated base. a) the sequence logo of the m4C methylations on cytosines; b) the m6A methylation of adenine.

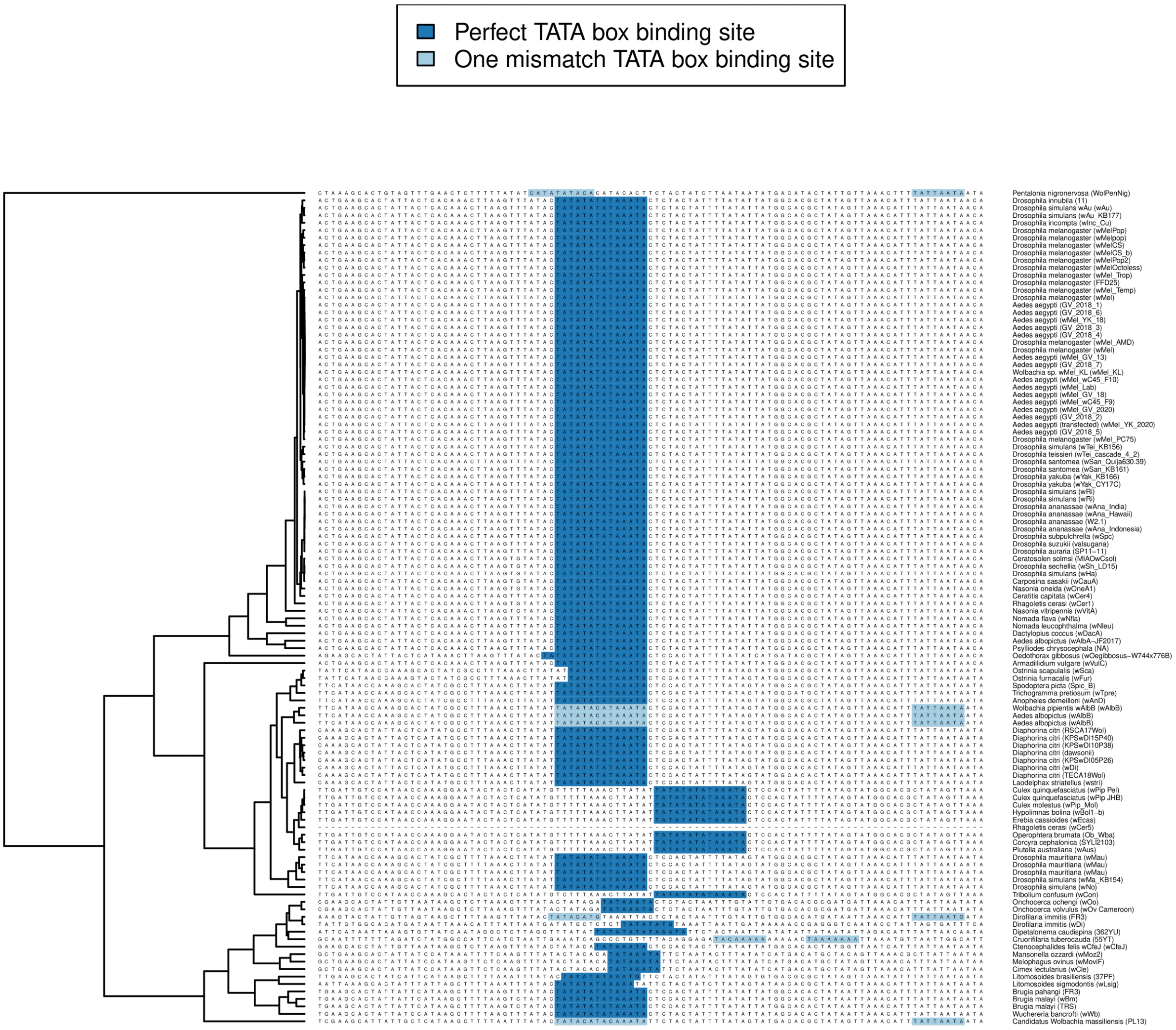

Figure S4. TATA boxes in the 100 bp regions upstream the *mid*A gene

The 100 bp regions upstream of the *mid*A gene on 111 representative Wolbachia strains are shown. On the left, the Maximum Likelihood (ML) phylogenetic analysis obtained from the concatenate of the single copy core genes. On the right the perfect TATA boxes in blue and one mismatched TATA boxes in azure. One sequence (GCF_018454445.1, *Wolbachia* endosymbiont of Rhagoletis cerasi - wCer5) is composed only by gap because the *mid*A gene was placed on the extreme of the contig and it was not possible to retrieve the upstream region.

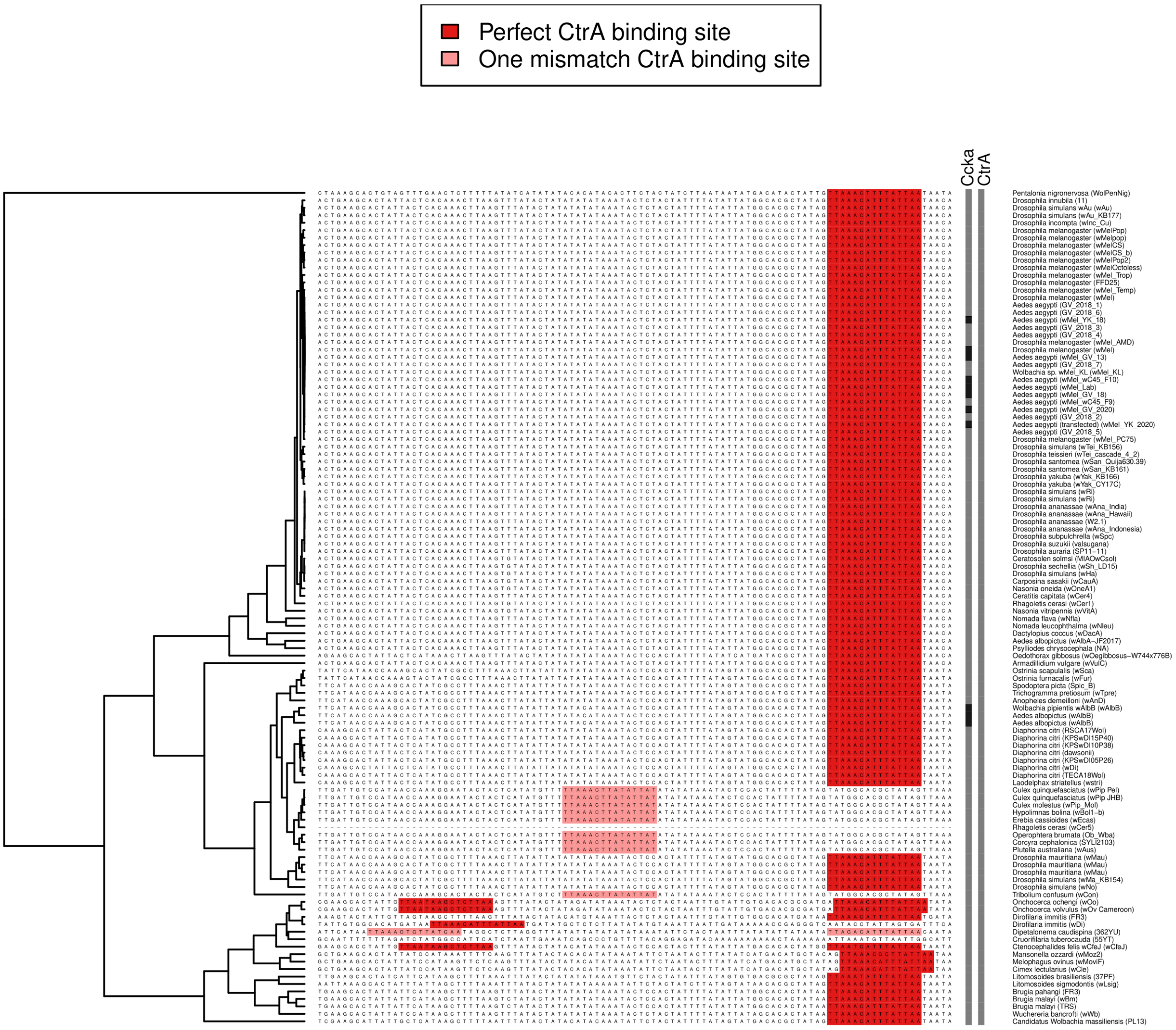

Figure S5. CtrA boxes in the 100 bp regions upstream the *mid*A gene

The 100 bp regions upstream of the *mid*A gene on 111 representative Wolbachia strains are shown. On the left, the Maximum Likelihood (ML) phylogenetic analysis obtained from the concatenate of the single copy core genes. On the right are the perfect CtrA boxes in red and one mismatched TATA boxes in light red. One sequence (GCF_018454445.1, *Wolbachia* endosymbiont of Rhagoletis cerasi - wCer5) is composed only by gap because the *mid*A gene was placed on the extreme of the contig and it was not possible to retrieve the upstream region.

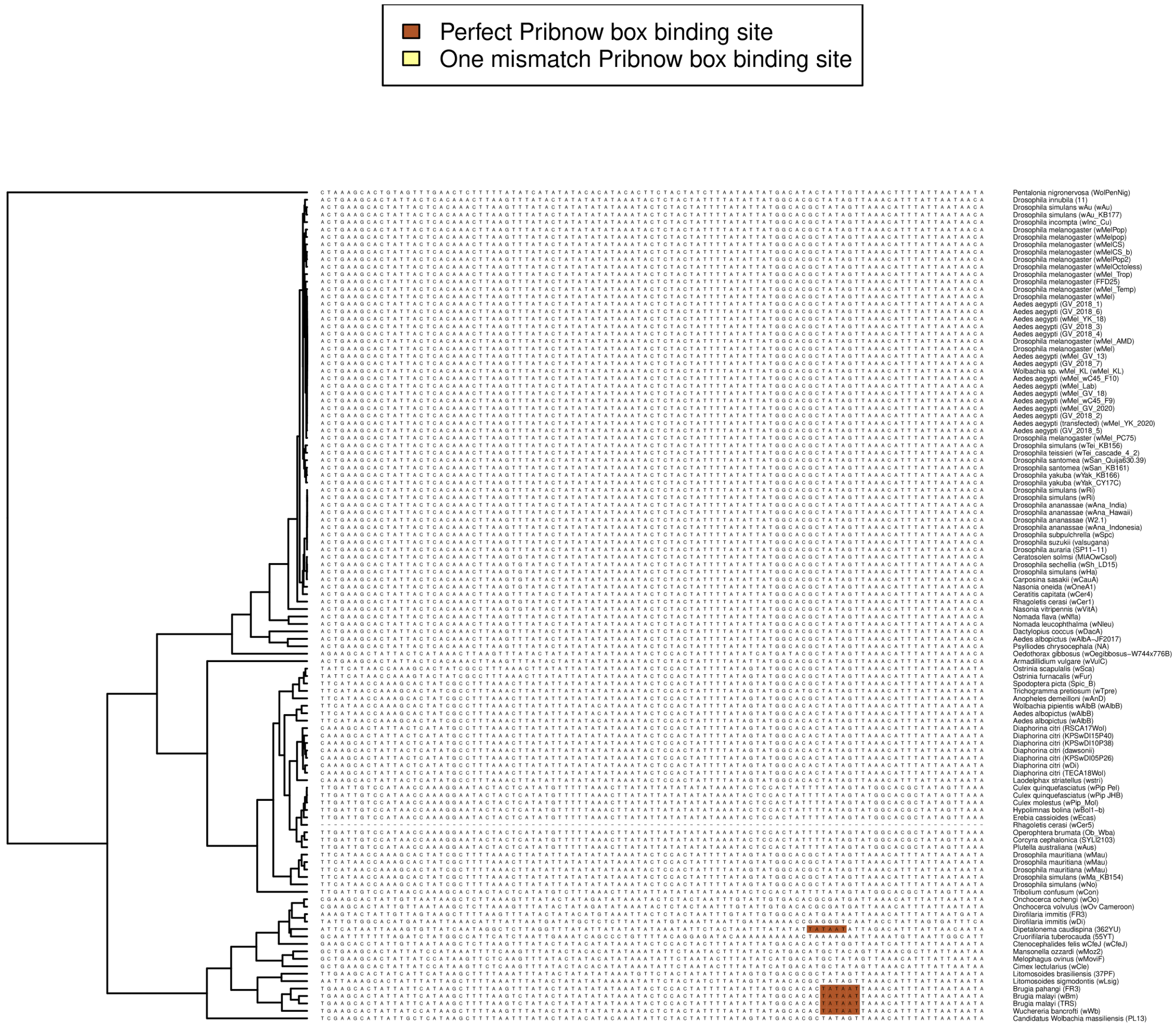

Figure S6. Pribnow boxes in the 100 bp regions upstream the *mid*A gene

The 100 bp regions upstream of the *mid*A gene on 111 representative Wolbachia strains are shown. On the left, the Maximum Likelihood (ML) phylogenetic analysis obtained from the concatenate of the single copy core genes. On the right the perfect Pribnow boxes in brown. One sequence (GCF_018454445.1, *Wolbachia* endosymbiont of Rhagoletis cerasi - wCer5) is composed only by gap because the *mid*A gene was placed on the extreme of the contig and it was not possible to retrieve the upstream region.

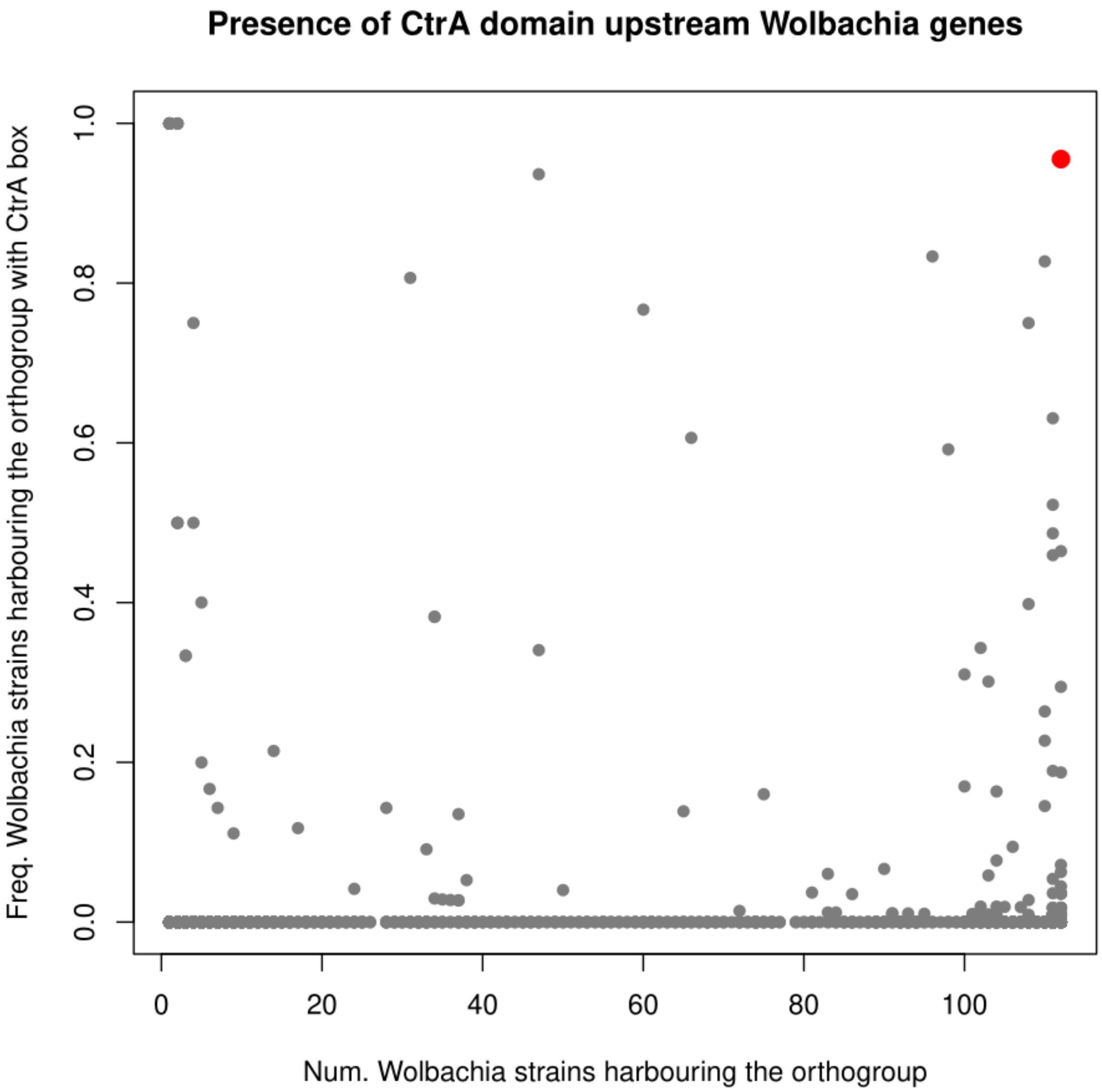

Figure S7. Scatter plot between ortholog gene and CtrA binding domains occurrence

Scatter plot in which each point is one of the ortholog genes found among the 112 *Wolbachia* genomes included in the study. On the x-axis, the number of *Wolbachia* genomes harbouring the ortholog gene and on the y-axis the frequency of sequences preceded by CtrA binding domain. The red dot corresponds to the *mid*A gene.

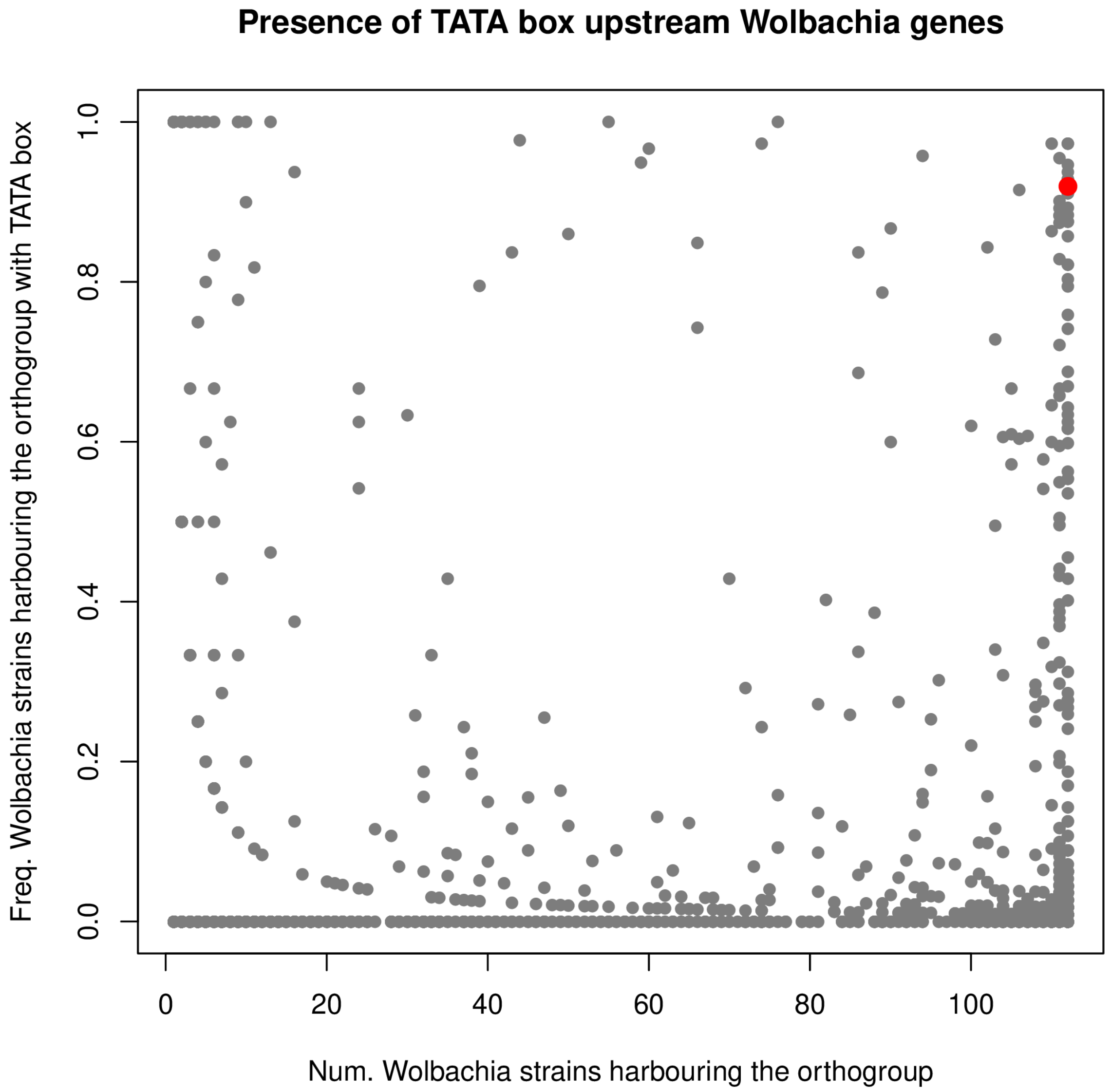

Figure S8. Scatter plot between ortholog gene and TATA box occurrence

Scatter plot in which each point is one of the ortholog genes found among the 112 *Wolbachia* genomes included in the study. On the x-axis, the number of *Wolbachia* genomes harbouring the ortholog gene and on the y-axis the frequency of sequences preceded by TATA box. The red dot corresponds to the *mid*A gene.

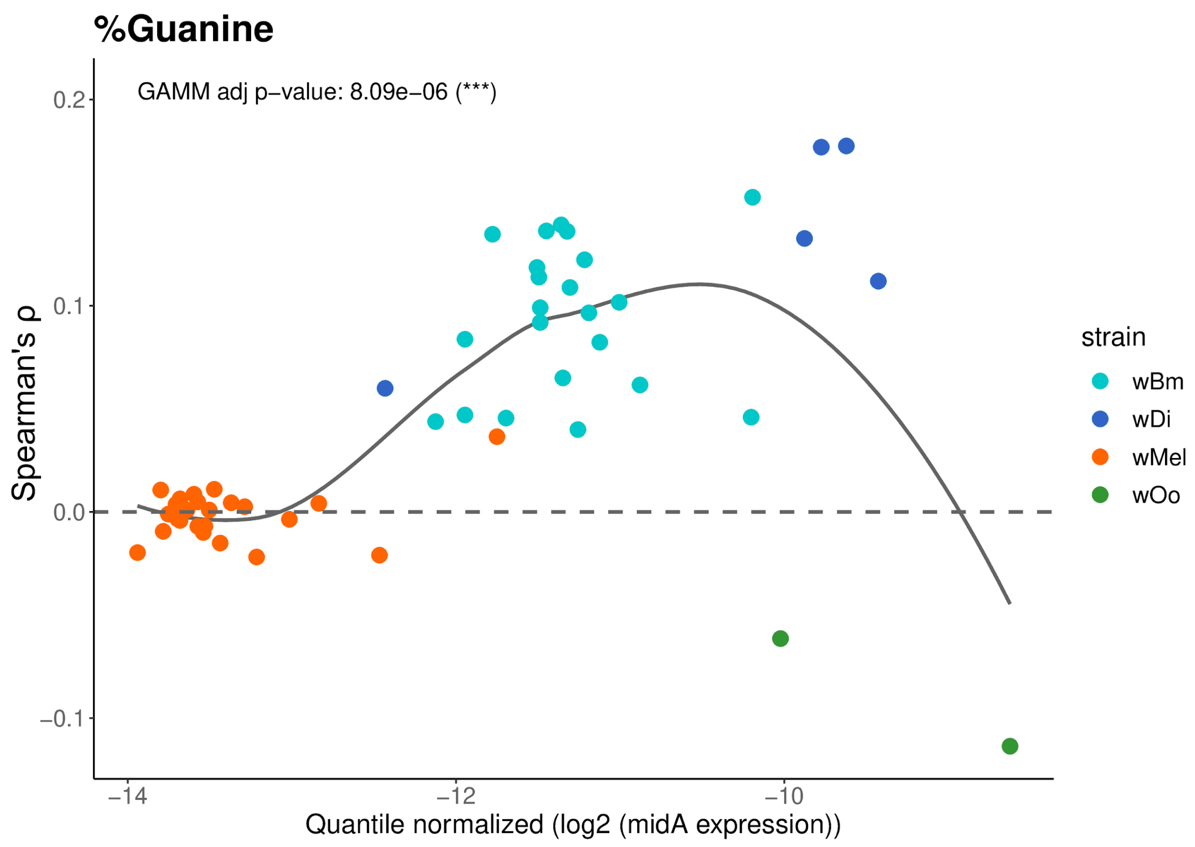

Figure S9. Correlation between midA expression and the association of percentage of guanine with gene expression

The scatter plot illustrates the relationship between *mid*A gene expression and the strength of association between percentage of guanine and gene expression across the 55 samples analyzed. Each point represents a sample and is color-coded by strain: azure for *w*Bm, blue for *w*Di, orange for *w*Mel, and green for *w*Oo. The x-axis displays the quartile-normalized $\log_2$ expression of midA, while the y-axis shows the Spearman's ρ value indicating the correlation between percentage of guanine and gene expression within that sample. The p-value from a generalized additive mixed model (GAMM), with strain included as a random effect, is reported below each plot.

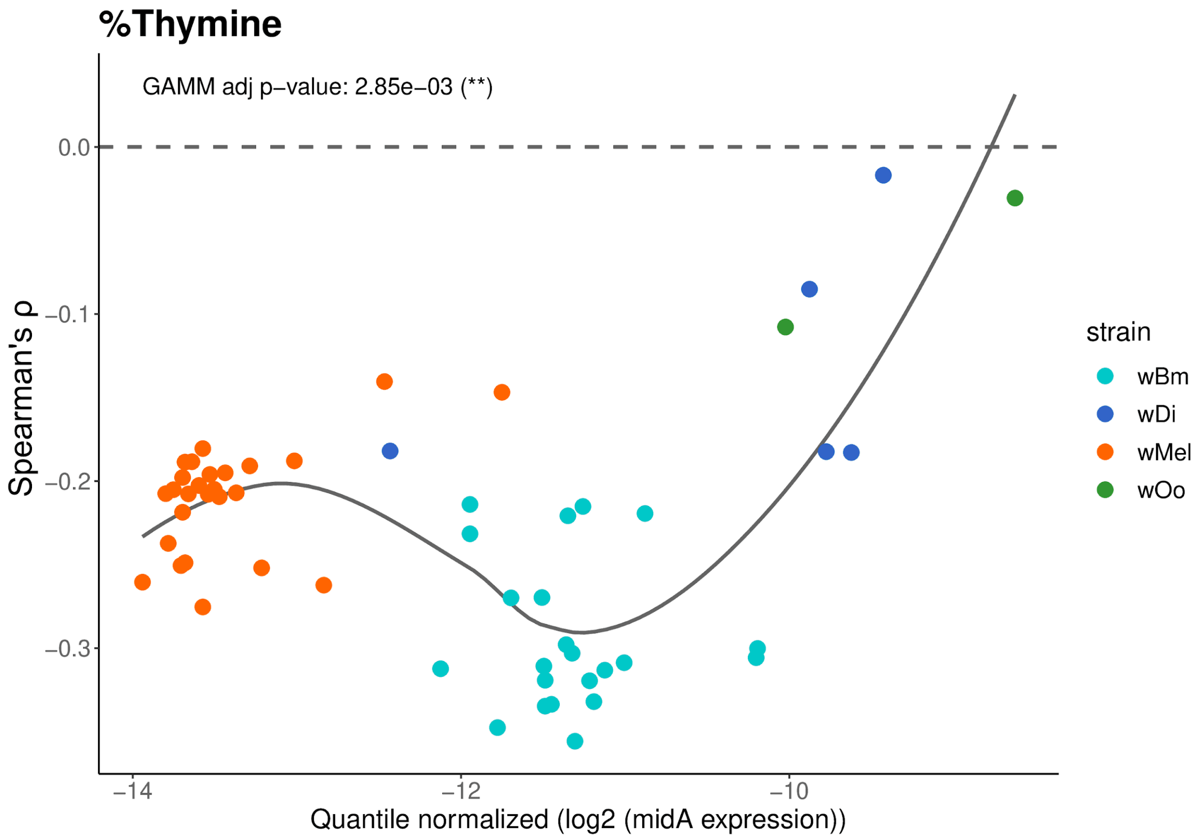

Figure S10. Correlation between midA expression and the association of percentage of thymine with gene expression

The scatter plot illustrates the relationship between *mid*A gene expression and the strength of association between percentage of thymine and gene expression across the 55 samples analyzed. Each point represents a sample and is color-coded by strain: azure for *w*Bm, blue for *w*Di, orange for *w*Mel, and green for *w*Oo. The x-axis displays the quartile-normalized log_2 expression of midA, while the y-axis shows the Spearman's ρ value indicating the correlation between percentage of thymine and gene expression within that sample. The p-value from a generalized additive mixed model (GAMM), with strain included as a random effect, is reported below each plot.

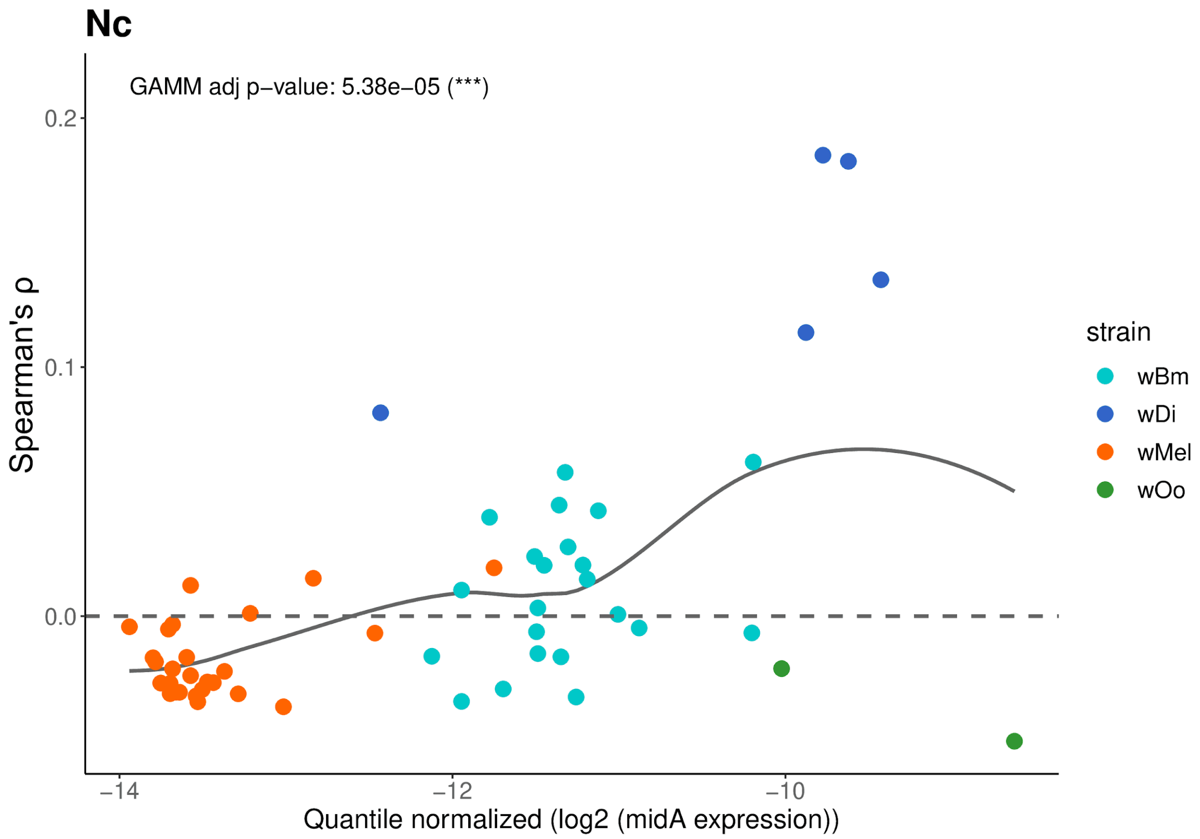

Figure S11. Correlation between midA expression and the association of the effective number of codons (Nc) with gene expression

The scatter plot illustrates the relationship between *mid*A gene expression and the strength of association between Nc and gene expression across the 55 samples analyzed. Each point represents a sample and is color-coded by strain: azure for *w*Bm, blue for *w*Di, orange for *w*Mel, and green for *w*Oo. The x-axis displays the quartile-normalized $\log_2$ expression of midA, while the y-axis shows the Spearman's ρ value indicating the correlation between percentage of Nc and gene expression within that sample. The p-value from a generalized additive mixed model (GAMM), with strain included as a random effect, is reported below each plot.

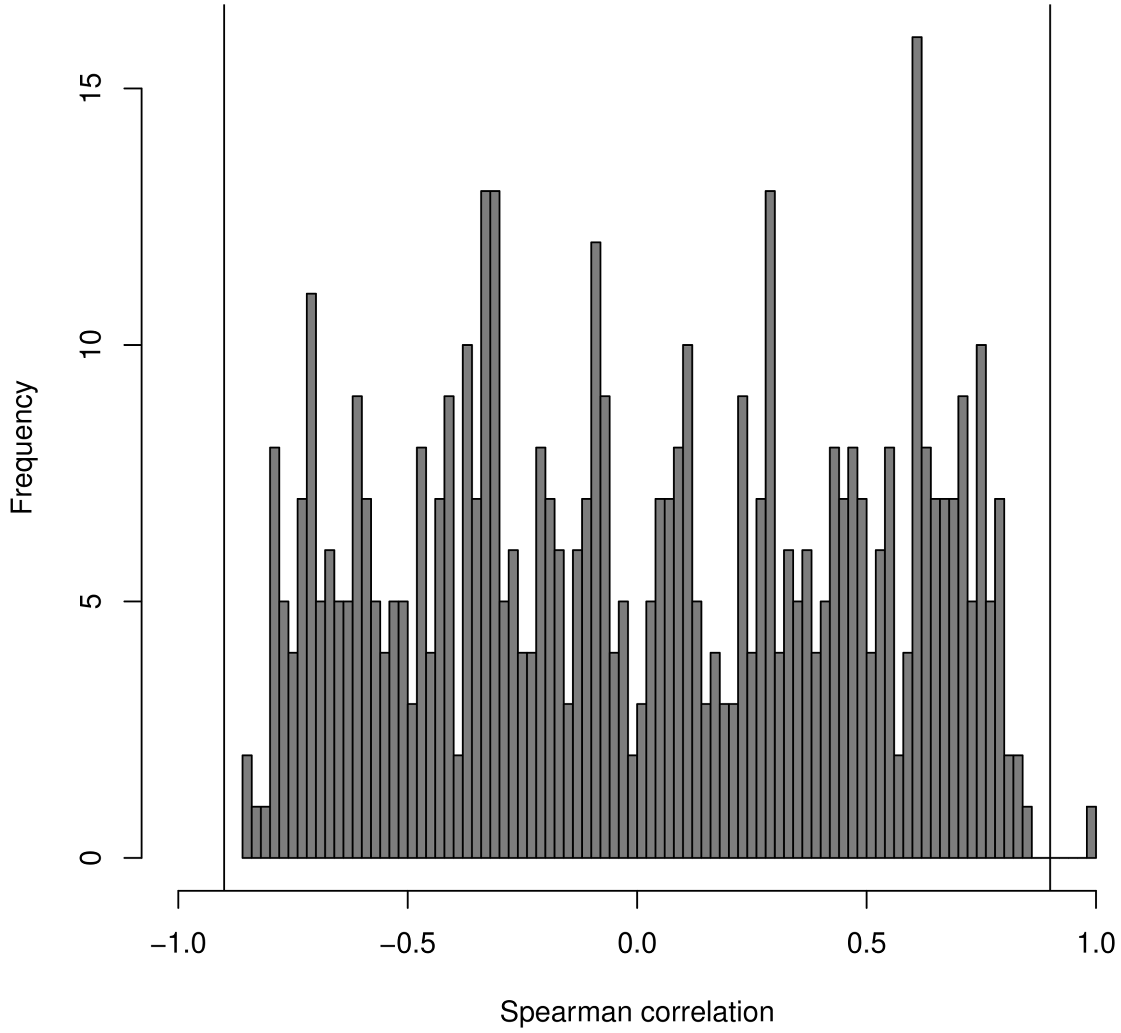

Figure S12. Distribution of the expression distances from *mid*A

The gene expression of all the core genes present among all the 55 samples included in the study were compared by Spearman co-correlation. The distribution of the distances from the *mid*A gene is reported. The vertical line identifies the -0.9 and 0.9 distance values. The correlation value 1 is due to the gene against itself.

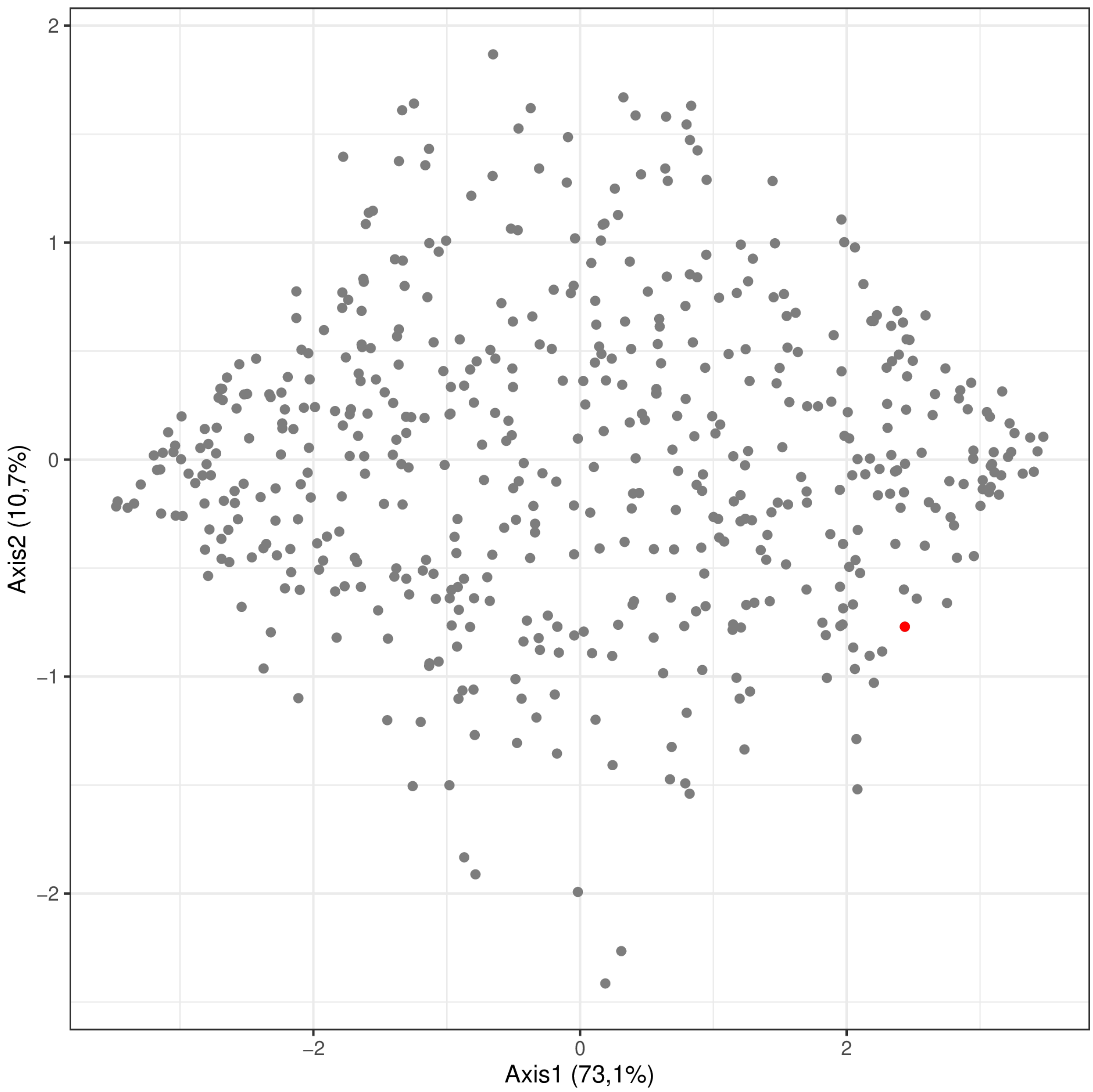

Figure S13. Principal Component Analysis (PCA) on the expression of all core genes

The gene expression of all the core genes present among all the 55 host-strain conditions included in the study were subjected to Principal Component Analysis (PCA) for the detection of gene, or cluster of gene, with an expression pattern similar to *mid*A (in red in the plot).

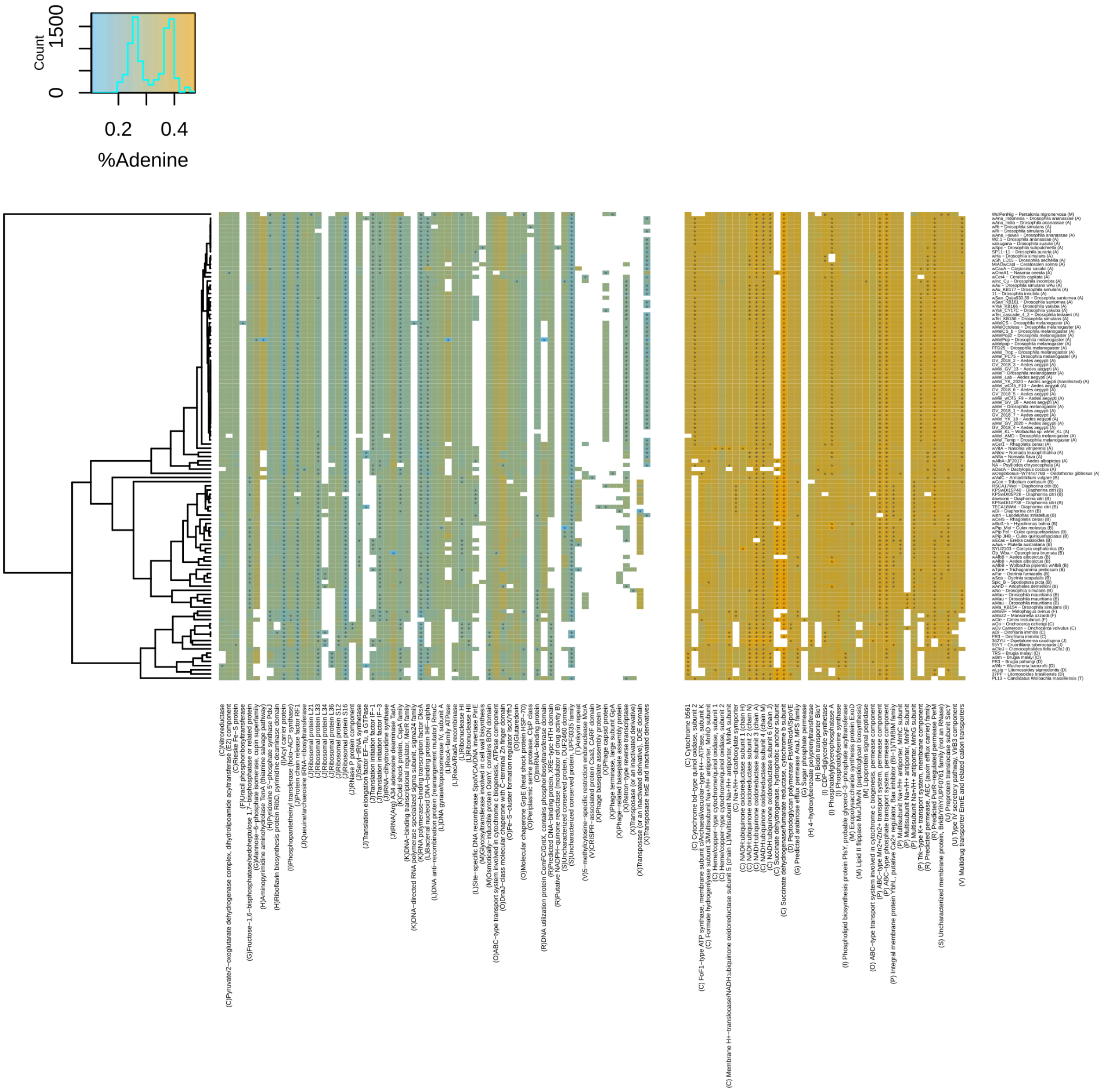

Figure S14. COG annotated genes with highest or lowest percentage of adenine

The heatmap shows the pattern of the genes with high or low C/A ratio among the 112 *Wolbachia* strains included in the study. On the left, the Maximum Likelihood phylogenetic tree obtained from concatenate of the single copy core genes. In the middle, heatmap reports the pattern of the 10 lower %A ratio COG annotated genes: rows refer to strains (matching with the phylogenetic tree on the left) and columns to genes (COG annotation is reported). Cell colours report the %A value following the legend on lower left. Asterisks are reported for genes among those in the 10 lower %A for the relative strain. More on the right, a heatmap reports the 10 higher %A COG annotated genes.

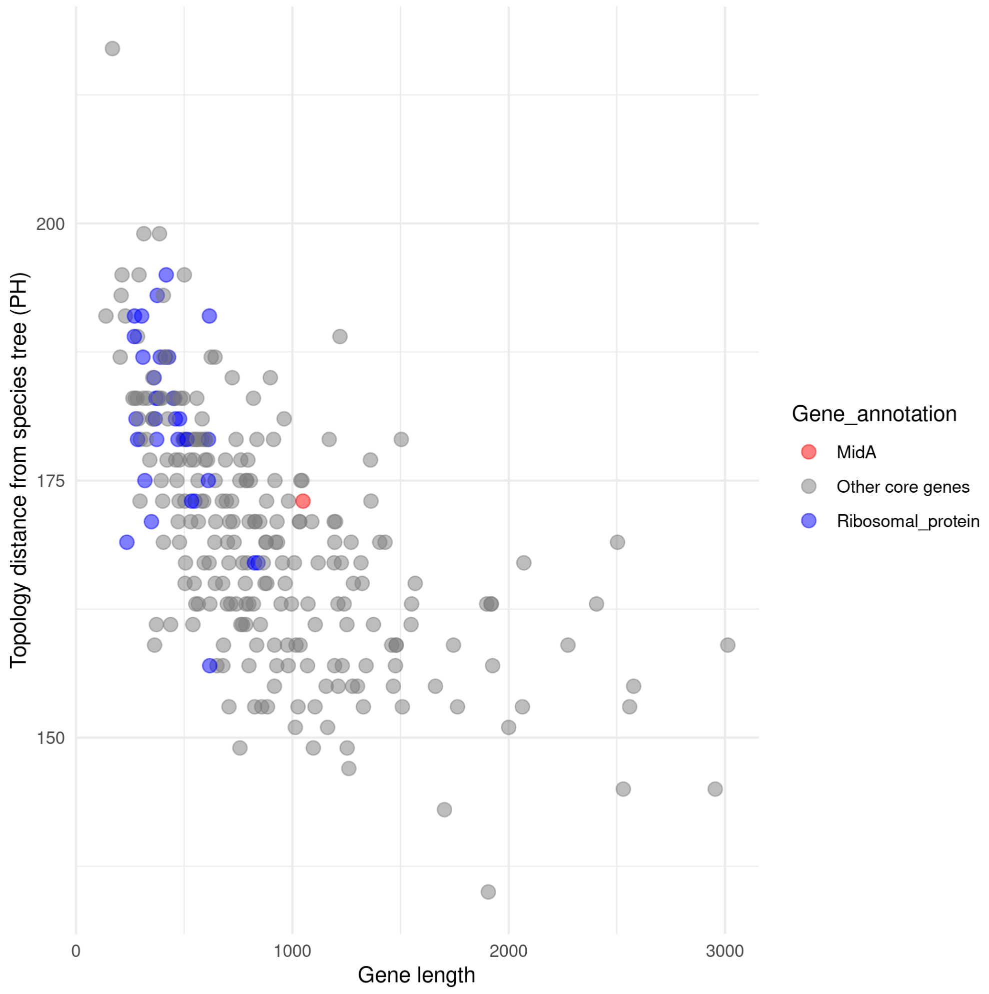

Figure S15: Relationship between gene length and phylogenetic congruence in *Wolbachia*.

This scatter plot displays the relationship between gene length (x-axis) and the Penny and Hendy tree distance (y-axis) between each of the 263 single-copy core gene trees and the *Wolbachia* species tree. Each point represents a single gene. Ribosomal protein genes are highlighted in blue, and the *MidA* gene is shown in red. The overall trend indicates that tree distance decreases with increasing gene length, likely because longer genes provide more phylogenetic signal. The *MidA* gene exhibits a tree distance comparable to both ribosomal and non-ribosomal genes, suggesting no strong evidence for horizontal gene transfer (HGT) affecting this gene.

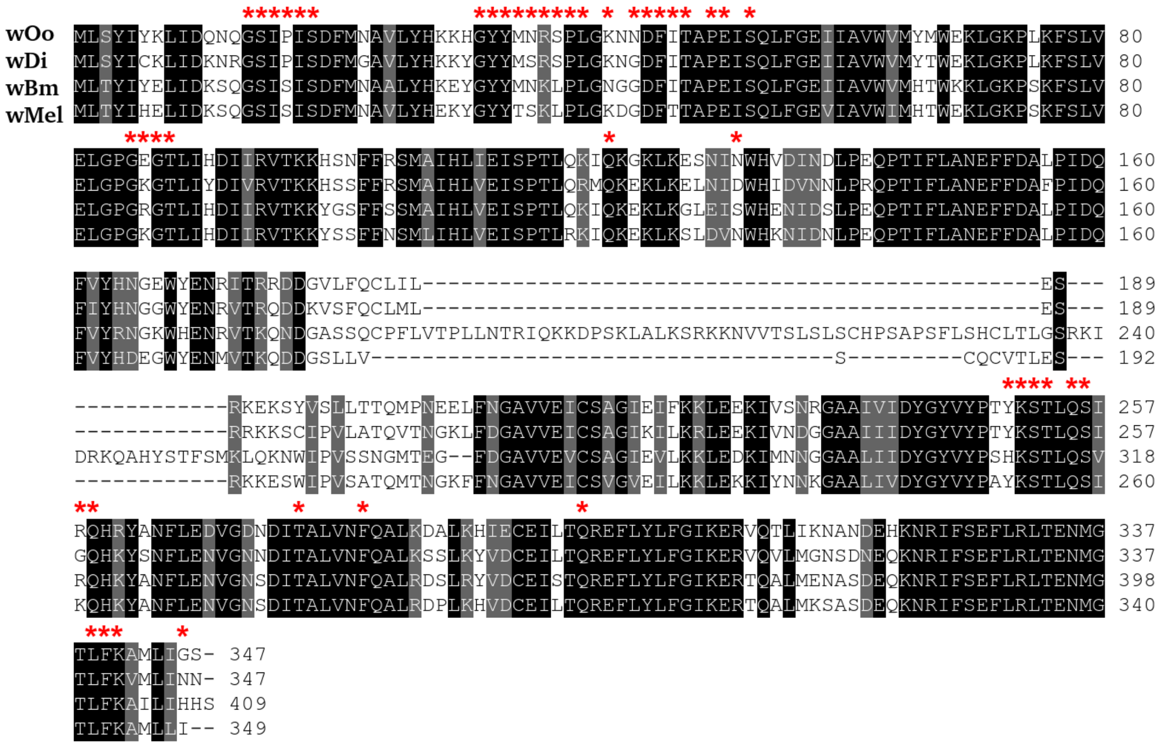

Figure S16. Alignment of MidA from *w*Oo, *w*Di, *w*Bm and *w*Mel

The alignment of MidA proteins from *w*Oo, *w*Di, *w*Bm, and *w*Mel *Wolbachia* strains is presented. Fully conserved residues are highlighted in black, residues with similar chemical properties are shown in gray, and non-conserved amino acids appear in white. Putative DNA-binding sites are indicated with red asterisks.
